# Analysis of Ultrahigh Apparent Mobility in Oxide Field‐Effect Transistors

**DOI:** 10.1002/advs.201801189

**Published:** 2019-01-25

**Authors:** Changdong Chen, Bo‐Ru Yang, Gongtan Li, Hang Zhou, Bolong Huang, Qian Wu, Runze Zhan, Yong‐Young Noh, Takeo Minari, Shengdong Zhang, Shaozhi Deng, Henning Sirringhaus, Chuan Liu

**Affiliations:** ^1^ State Key Lab of Opto‐Electronic Materials & Technologies, Guangdong Province Key Lab of Display Material and Technology, School of Electronics and Information Technology, Shunde International Joint Research Institute Sun Yat‐Sen University Guangdong 510275 China; ^2^ Shenzhen Key Lab of Thin Film Transistor and Advanced Display, Peking University Shenzhen Graduate School Peking University Shenzhen 518055 China; ^3^ Department of Applied Biology and Chemical Technology The Hong Kong Polytechnic University Hung Hom Kowloon Hong Kong SAR; ^4^ The Hong Kong Polytechnic University Shenzhen Research Institute Shenzhen 518057 China; ^5^ Department of Energy and Materials Engineering Dongguk University 30 Pildong‐ro, 1 gil, Jung‐gu Seoul 04620 Republic of Korea; ^6^ International Center for Materials Nanoarchitectonics (WPI‐MANA) National Institute for Materials Science (NIMS) Tsukuba Ibaraki 305‐0044 Japan; ^7^ Department of Physics University of Cambridge Cambridge CB3 1HK UK

**Keywords:** carrier mobility, doping, four‐probe measurement, surface potential scanning, thin‐film transistors

## Abstract

For newly developed semiconductors, obtaining high‐performance transistors and identifying carrier mobility have been hot and important issues. Here, large‐area fabrications and thorough analysis of InGaZnO transistors with enhanced current by simple encapsulations are reported. The enhancement in the drain current and on–off ratio is remarkable in the long‐channel devices (e.g., 40 times in 200 µm long transistors) but becomes much less pronounced in short‐channel devices (e.g., 2 times in 5 µm long transistors), which limits its application to the display industry. Combining gated four‐probe measurements, scanning Kelvin‐probe microscopy, secondary ion mass spectrometry, X‐ray photoelectron spectroscopy, and device simulations, it is revealed that the enhanced apparent mobility up to several tens of times is attributed to the stabilized hydrogens in the middle area forming a degenerated channel area while that near the source‐drain contacts are merely doped, which causes artifact in mobility extraction. The studies demonstrate the use of hydrogens to remarkably enhance performance of oxide transistors by inducing a new mode of device operation. Also, this study shows clearly that a thorough analysis is necessary to understand the origin of very high apparent mobilities in thin‐film transistors or field‐effect transistors with advanced semiconductors.

In recent studies of advanced semiconductors, obtaining high‐performance transistors and identifying carrier mobility have been hot and important issues. For example, amorphous oxide semiconductors (AMOS)^1^ feature superior performance over amorphous silicon in terms of high mobility and good stability,^2^ regarded as the next generation of backplane for flat panel displays^3^ or sensor circuits.^4^ The mobility of AMOS, e.g., amorphous InGaZnO (a‐IGZO), is usually 10–40 cm^2^ V^−1^ s^−1^ in thin‐film transistors (TFTs) and cannot compete with polycrystalline silicon in many applications^5^ such as functional logic circuits. However, there have been efforts that lead to higher apparent mobility values in transistors by incorporating nanomaterials, micropatterning, multiple‐active layers, etc. The use of silver nanowires or single‐wall carbon nanotubes was demonstrated to achieve a high mobility of 174^6^ and 120 cm^2^ V^−1^ s^−1^,^7^ respectively. In addition, partial treatment of the active layers, e.g., by nanometer Ar plasma treatment, fine‐patterning microbelts,^8^ or placing a capping metal on top of IGZO, demonstrate mobility of 79^9^ and 160 cm^2^ V^−1^ s^−1^,^10^ respectively. Yet to induce conducting materials or areas partially into channels may require complicated fabrications or long relaxation time (in several days).^10^ These values are already higher than that of single‐crystalline IGZO film, i.e., about 80 cm^2^ V^−1^ s^−1^.^11^ The discrepancy raises two important and urgent questions: 1) Is it possible to use simple techniques to obtain oxide transistors with very high apparent mobility? 2) How to analyze and understand the transistors with very high apparent mobility?

Here, we present that IGZO TFTs with simple and optimized encapsulations can achieve significantly enhanced on–off ratio as compared with pristine IGZO TFT. The long‐channel (200 µm) devices show tens of times higher on‐current and apparent field‐effect mobility, while in short‐channel devices such an effect becomes minor. The devices were fabricated by simply depositing top encapsulation layers via plasma enhanced chemical vapor deposition (PECVD) at a relatively low temperature. To understand the origin of enhanced current and why it is only significant in long‐channel devices, we investigated the current–voltage characteristics, capacitance–voltage relations, gated four‐probe measurement (GFP) for potentials, scanning Kelvin probe microscope (SKPM) for surface, 2D technology computer aided design (TCAD) device simulations, depth‐profiling of elements, photoelectron spectroscopy for ions, density functional theory (DFT) calculations, and temperature‐dependent measurements. The various investigations give highly consistent understandings for the origin of ultrahigh apparent mobility, demonstrate the necessity of critical analysis for high‐performance TFT or field‐effect transistor (FET), and provide a partially doped device structure with enhanced on–off ratio and operational reliability.

The IGZO film was deposited following the regular routine to form a TFT in the bottom‐gate, top‐contact (BGTC) configuration. Then SiO*_X_* (350 nm) and SiN*_X_* (100 nm) layers were consequently deposited by PECVD at a relatively low temperature (150 °C). To obtain high on–off ratios, it is very important to use the relatively low temperature PECVD and to deposit the SiO*_X_* and SiN*_X_* films with the optimum conditions to reach the mentioned thickness. The device structure and optical images are shown in **Figure** **1**. Note that channel length of these devices was patterned to be 200 µm. For direct comparisons, only the upper part of the arrays was covered by SiN*_X_*/SiO*_X_* encapsulation layers (referred as IGZO‐H film in the following), while the rest are the pristine IGZO TFTs. The two types of TFTs exhibit distinct measured transfer characteristics (Figure 1c), where drain current (*I*_D_) is 40 times higher in the treated TFTs in the linear regime scanning. The hysteresis is small and the output characteristics are shown in Figure 1d. The average field‐effect mobility is extracted according to the literatures of oxide TFTs.^5^, ^12^ In the linear regime (*V*_D_ = 0.1 V), *µ*_lin_ extracted by the slope of *I*_D_ against *V*_GS_ 12 is 345 cm^2^ V^−1^ s^−1^ (Figure 1e), as compared with 8.0 cm^2^ V^−1^ s^−1^ in regular IGZO devices. The values of threshold voltage *V*_TH_ and subthreshold swing (SS) are similar for the two types of TFTs. In the saturated regime (*V*_D_ = 20 V), the average mobility extracted by the slope of $\sqrt{I_{D}}$ is 402 cm^2^ V^−1^ s^−1^ (Figure 1f). The differential mobility in the linear regime is extracted as, *µ*_FE_ = (*L*/*WC*_i_ *V*_DS_)(∂*I*_D_/∂*V*_GS_), where *C*_i_ is the gate insulator capacitance per unit area and the *µ*_FE_–*V*_GS_ curves are presented in Figure 1g,h. The mobility rises gradually with the increase of *V*_GS_ following the percolation theory.^13^ For IGZO‐H TFT, *µ*_FE_ rises to a peak value of 384 cm^2^ V^−1^ s^−1^ at *V*_GS_ is set at 10.75 V, and decreases to 314 cm^2^ V^−1^ s^−1^ when *V*_GS_ is set at 20 V, somehow similar to that in polycrystalline or single crystalline silicon TFTs where surface scattering dominates at a high gate‐field.^13^ The reliability factors of mobility^14^ for the saturated and linear regime are calculated as $r_{\mathrm{sat}}={\left((\sqrt{I_{\mathrm{Dmax}}}-\sqrt{I_{D0}})/V_{\mathrm{Gmax}}\right)}^{2}/{\left(WC_{i}\mu_{\mathrm{sat}}/2L\right)}_{\mathrm{claimed}}$ and *r*_lin_ = ((*I*_Dmax_ − *I*_D0_)/*V*_Gmax_)/(*V*_DS_ *WC*_i_μ_lin_/*L*)_claimed_. Here, *I*_Dmax_ and *I*_D0_ are the current at *V*_GS_ = 20 and 0 V, respectively. The values are 83.6% (saturated) and 78.2% (linear) for IGZO‐H TFT, whereas those of IGZO TFT are 68.5% (saturated) and 79.0% (linear), respectively.

**Figure 1:**
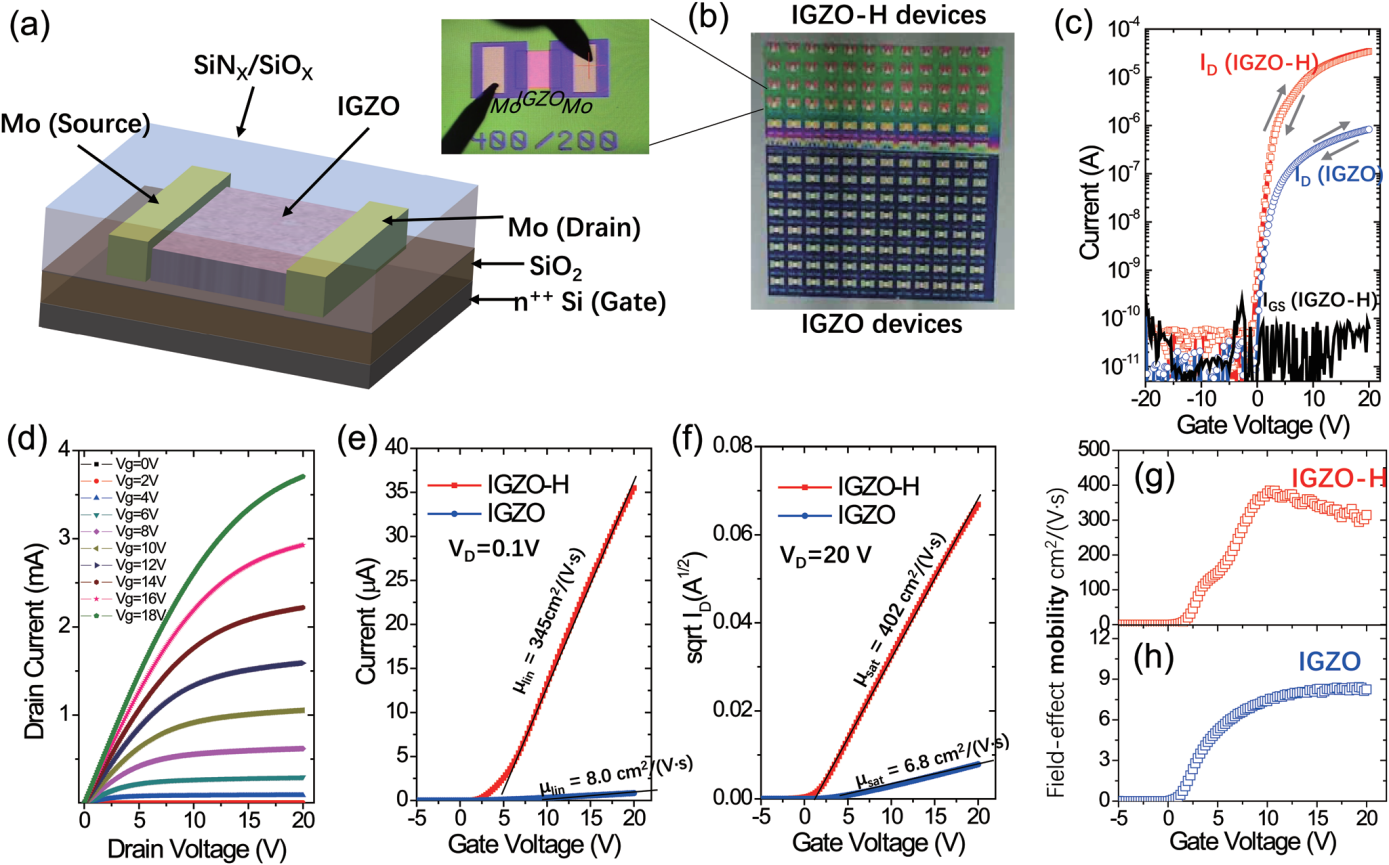
Characterizations of TFTs. a) Schematic view of a TFT device. b) Optical image of TFT arrays with the upper half IGZO‐H and the lower half remains pristine, where one of the devices is shown on the left. c) Transfer characteristics (*V*_D_ = 0.1 V) of IGZO‐H (red) and IGZO TFTs (blue) measured on the same substrates shown in (b). d) Output characteristics for various *V*_GS_ values. e) Transfer characteristics in the linear regime (*V*_D_ = 0.1 V) of IGZO‐H TFT and IGZO TFT, with the extracted linear mobility. f) Square root of drain current in the saturated regime (*V*_D_ = 20 V) IGZO‐H TFT (red) and IGZO TFT (blue), with the extracted saturated mobility. Extracted field effect mobility plotted against gate voltage of IGZO‐H TFT g) and IGZO TFT h) in the linear regime (*V*_D_ = 0.1 V).

To exclude errors in calculating the field‐effect mobility values, the capacitance of gate‐dielectric of the IGZO and IGZO‐H TFTs was measured and compared with that measured in the metal‐insulator‐metal (MIM) structure. As all of them show the same values when the devices are turned on (Figure S1, Supporting Information), miscalculation in capacitance^15^ can be excluded. Also, the direct comparison of current of the two types of devices exclude the probability of overestimation caused by gated Schottky injection.^16^ In those TFTs with serious Schottky barriers, drain current values would be small and some would exhibit two segments with very different slope values appear in the $\sqrt{I_{D}}$–*V*_G_ curves in the saturated regime (the so‐called “kink” feature).[[qv: 16a]] The relationships between differential field‐effect mobility against *V*_GS_ in Figure 1g,h (linear regime) and Figure S2 in the Supporting Information (saturated regime) show only small decrease at high *V*_GS_ and the simulations using the high mobility values fit the transfer and output curves well (Figures S3 and S4, Supporting Information). The IGZO‐H TFTs show better stability in bias‐stressing than IGZO TFTs do (Figure S5, Supporting Information) and the statistical information is shown in Figure S6 (Supporting Information).

The remarkable enhanced current comes from the encapsulation layers and postannealing, which was systematically studied and is probably related to moderate hydrogen diffusion from the top layers into IGZO. First, we deposited the SiO*_X_* layer only, SiN*_X_* layer only, or SiO*_X_*/SiN*_X_* bilayer on IGZO TFTs with the same postannealing (**Figure** **2**a). The measured results evidence that the high current from the SiN*_X_* layer but not from SiO*_X_* layer (see Figure 2a). This is probably because PECVD‐grown SiN*_X_* (using SiH_4_ and NH_3_ gas flows) contains higher concentration of hydrogen than PECVD‐grown SiO*_X_* (using SiH_4_ and N_2_O gas flows).^17^ The function of SiO*_X_* layer is to mainly improve the subthreshold behaviors and to reduce SS (see Figure 2a and more details in Figures S7–S9, Supporting Information). Second, the device performance significantly relies on postannealing with SiN*_X_* film. In a control experiment, after PECVD deposition of SiO*_X_*/SiN*_X_* layer, the SiN*_X_* film was etched before postannealing. The resulting TFT exhibits similar performance with pristine IGZO TFT (Figure S10, Supporting Information), confirming that the high capacity of gate‐tuning in conductance comes from annealing with SiN*_X_* layer. In addition, we compared the devices with SiN*_X_* films in different thicknesses as shown in Figure 2b (also in Figure S11, Supporting Information). The device with 20 nm SiN*_X_* (black) merely shows improvement in the current, whereas that with 100 nm SiN*_X_* (blue) shows two magnitudes higher current. In comparison, the devices with 300 nm (red) cannot be turned off. Therefore, it is possible to control the on–off ratio in devices by controlling the thickness of SiN*_X_* layer. It is also consistent with the proposed explanation that hydrogen diffusion during the moderate annealing process with ion sources can be the origin of the improved device performance.

**Figure 2:**
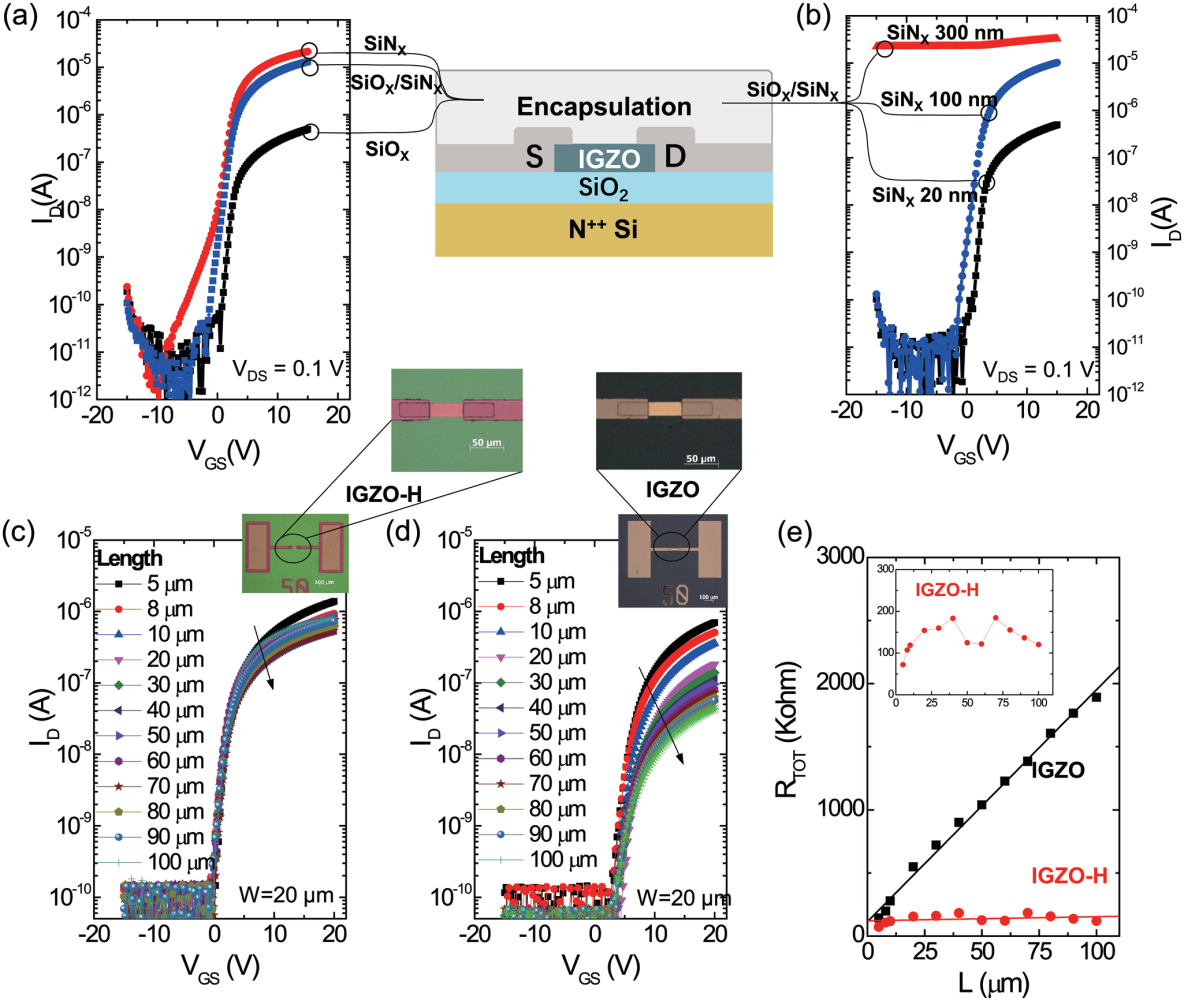
Effect of encapsulation layers and channel length scaling. a) Linear transfer curves with encapsulation layer of SiO*_X_* (350 nm, black), SiN*_X_* (100 nm, red), or SiO*_X_*/SiN*_X_* (350/100 nm, blue). b) Linear transfer curves with encapsulation layer of SiO*_X_*/SiN*_X_* with varying the thickness of SiN*_X_*: 20 nm (black), 100 nm (blue), or 300 nm (red). All the devices were annealed at 350 °C for 1 h after the final deposition. Transfer characteristics (*V*_DS_ = 0.1 V) of IGZO‐H c) and IGZO d) TFTs with channel length *L* varying from 5 to 100 µm (*W* = 20 µm) with optical images. The zoomed‐in images show the channel area with the semiconductor islands is narrower than the electrodes, avoiding the fringe current. e) Total resistance *R*_TOT_ against *L* for IGZO TFTs (black) and IGZO‐H TFTs (red), with the latter enlarged as inset. The dots are measured data and the lines are linear fittings.

Then the scaling effect for channel length of TFTs was investigated and devices with channel length *L* varying from 5 to 100 µm were characterized with *V*_DS_ as 0.1 V (Figure 2c–e). The semiconductor islands are narrower than the electrodes, avoiding the fringe current (Figure 2c,d, inset). The total resistance (*R*_TOT_ =*V*_D_/*I*_D_) at *V*_GS_ = 20 V is plotted against *L* (Figure 2e). Surprisingly, *R*_TOT_ of IGZO‐H device does not scale with *L* like that of IGZO TFT. Contact resistance *R*_C_ (the intercept from linear fitting of *R*_TOT_) of IGZO TFTs is about 114 kΩ and is similar with *R*_TOT_ of IGZO‐H TFTs. These results suggest that, for IGZO‐H devices, the resistance of the contact area and not that of the channel area is dominant in determining *R*_TOT_. Also, the increase in current is limited to large‐channel devices but not significant in small‐channel devices (Figure 2c–e). For instance, the increase in current for source–drain spacings of 5 µm is only in a factor of 2. Therefore, the benefits for the applications with short channel length (2–4 µm), such as ultrahigh‐resolution display backplane, will be limited at this stage.

To specifically investigate the channel area, we have built TFTs in the GFP structure (**Figure** **3**). During transfer characterizations, the potential drop between the two additional probes was measured as (*V*_XX_ = *V*_B_ − *V*_A_)^18^ with the distance *L*_XX_ between them. The two probes extend slightly into the channel with only 10 µm, much shorter than *W* of 400 µm (Figure 3a,e). For IGZO TFT, when *V*_GS_ is above 5 V in transfer scanning (*V*_DS_ = 0.1 V), *V*_A_ and *V*_B_ remained constant (Figure 3f), because the accumulated carrier concentration is uniform along the channel and the potentials are almost independent on *V*_GS_. The potential drop *V*_XX_ saturates and well scales with the distances between the probes at large *V*_GS_, i.e., *V*_XX_ ≈*V*_DS_ × *L*_XX_/*L*. In comparison, for IGZO‐H TFT, *V*_A_ and *V*_B_ gradually increase (Figure 3b), suggesting the carrier concentration distribution is not uniform and the potentials change with the increase of *V*_GS_. Also, we found the potential drop *V*_XX_ ≪ *V*_DS_ × *L*_XX_/*L* and *V*_XX_ continually increases with the increase of *V*_GS_, indicating a highly conductive channel. If using the potentials to extract the channel field‐effect mobility by *µ*_FE‐CH_ = (*L*_XX_/*WC*_i_ *V*_XX_)(∂*I*_D_/∂*V*_GS_),^19^ the extracted values for IGZO TFT is close to *µ*_FE_ extracted from transfer curves (Figure 3h). However, the extracted *µ*_FE‐CH_ of IGZO‐H TFT is above 1000 cm^2^ V^−1^ s^−1^, much higher than *µ*_FE_ extracted from transfer curves in TFTs. The small voltage drop between the two voltage probes suggests that most of the channel area is degenerately doped and highly conducting, and the current is limited by the resistance of short, less conducting regions near the contacts. Thus, the standard mobility extraction from four‐probe measurements is not applicable for the IGZO‐H TFT.

**Figure 3:**
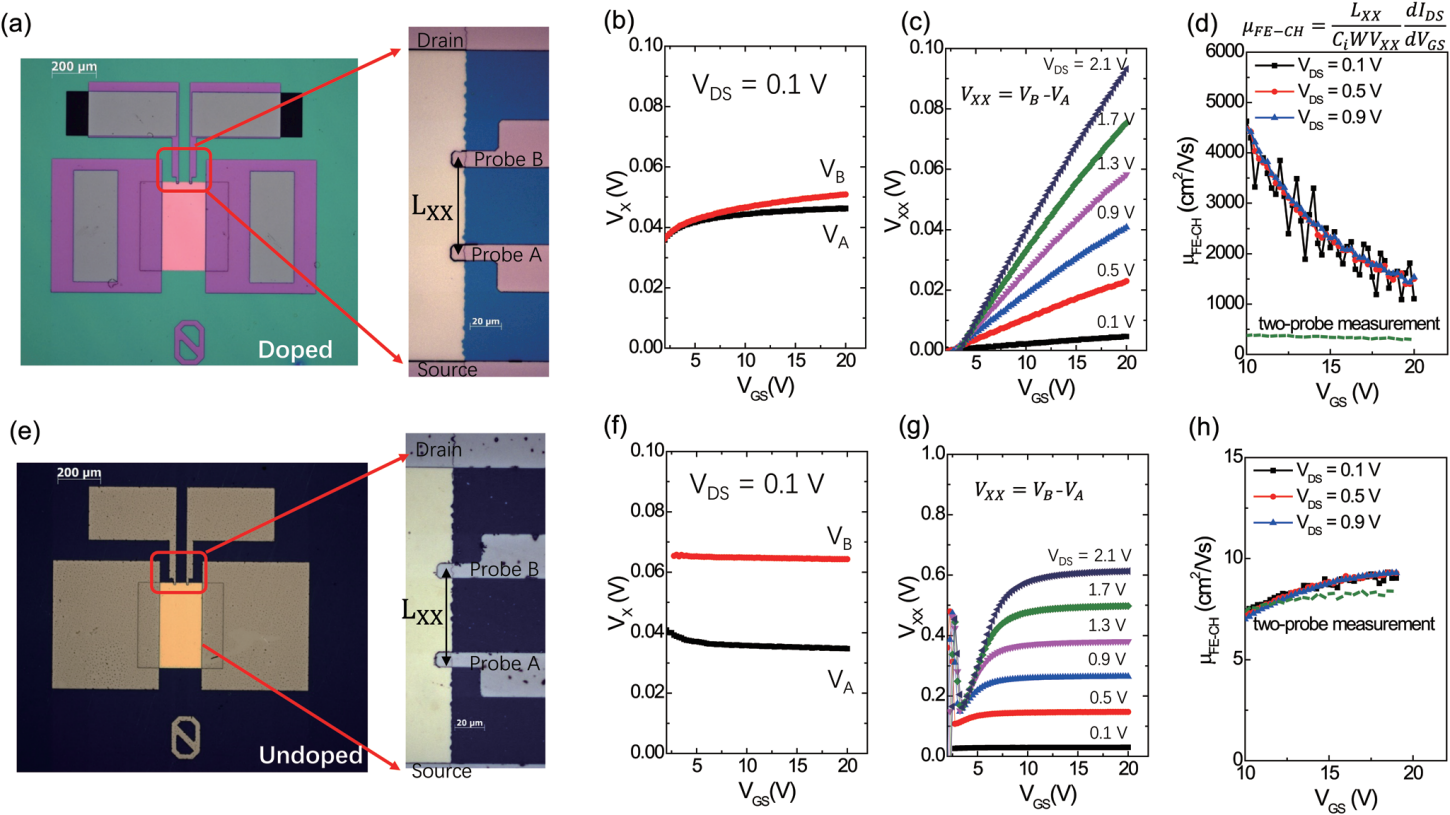
Gated four‐probe (GFP) measurements. a) Optical image of one IGZO‐H TFT and the region near the probes is enlarged on the right. b) Potential of probe A and B in IGZO‐H TFT with *V*_DS_ = 0.1 V. c) Potential drop *V*_XX_ = *V*_B_ −*V*_A_ as a function of *V*_GS_ with various *V*_DS_. d) The extracted channel field‐effect mobility of IGZO‐H TFT with various *V*_DS_ by GFP measurements (dots) or transfer curves of TFTs (dashed lines). The corresponding measurements of IGZO TFT are shown in (e–h).

Accordingly, we propose that the ultrahigh apparent mobility is not a reflection of a genuine high quality semiconducting channel, but results from a highly doped channel region gapped by short, more resistive regions near the contacts (**Figure** **4**a). Essentially, the device operates with a much shorter effective channel length than the distance between the source–drain electrodes. The high level of n‐type doping in the channel region could be caused by H‐doping during the deposition of the encapsulation layer, where hydrogen atoms diffuse from SiN*_X_* into the IGZO channel. Near and underneath the Mo electrodes, this hydrogen diffusion may be blocked or hydrogen may be adsorbed by the Mo giving rise to more resistive contact regions. Actually, it has been proven that most of metals including Mo easily adsorb hydrogens.^20^ The proposed scenario is supported by another multiprobe measurement where the inner probes are long enough to cross the channel (see Figures S14–S15 Supporting Information for the analysis.).

**Figure 4:**
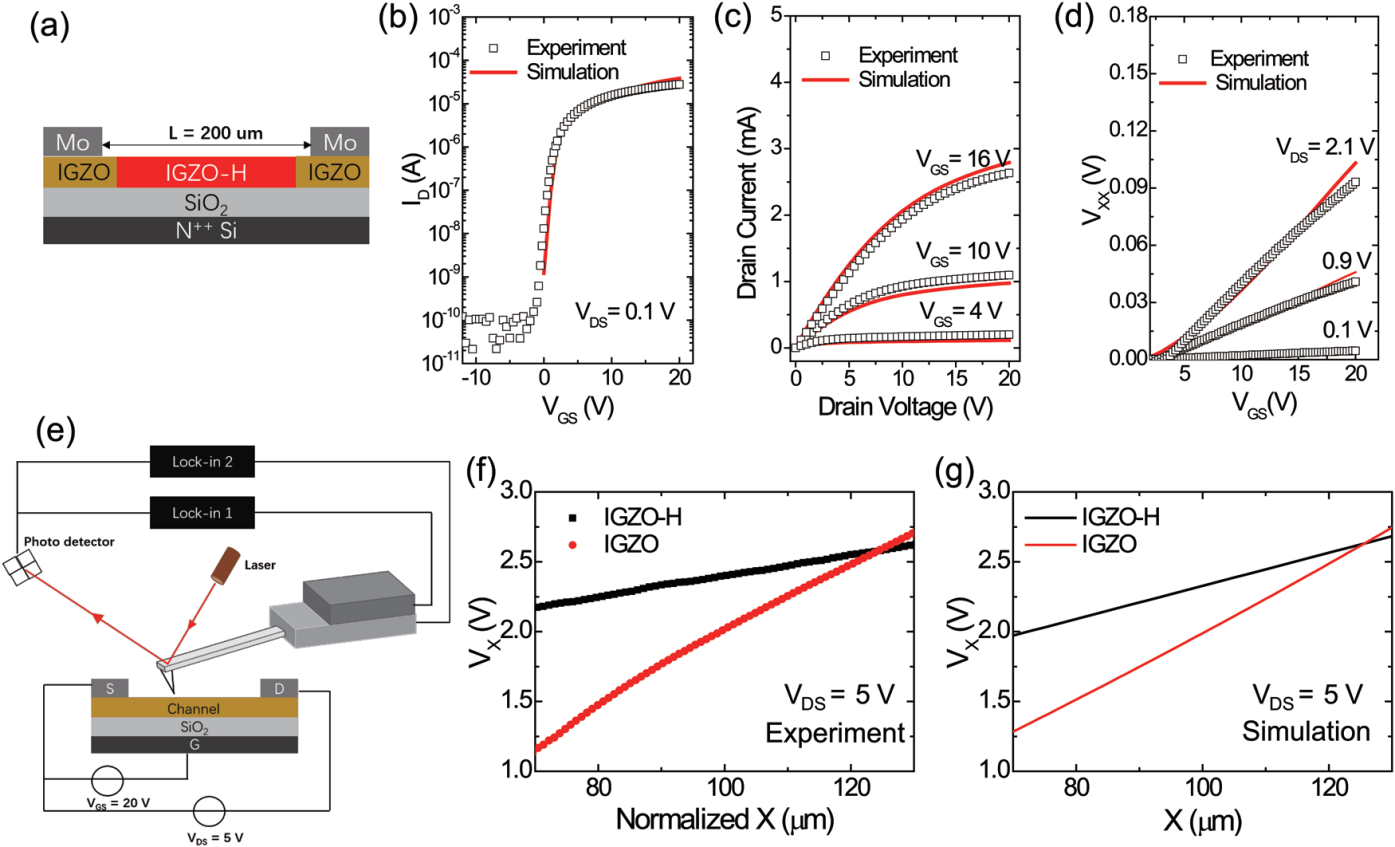
Simulations and scanning Kelvin probe microscope (SKPM) measurements. a) The proposed structure model, the red area represents doped region with high carrier concentration and the blond area refer to pristine IGZO with no doping. b) The transfer characteristics in linear regime (*V*_DS_ = 0.1 V). c) Output curves. d) Simulated potential drops *V*_XX_ under various *V*_DS_ bias set. e) Schematic view of SKPM measurement. f) Measured and g) simulated results of potential along the channel between probe A and B for both TFTs.

The proposed mechanism is further examined by 2D TCAD device simulations and SKPM measurements. For pristine IGZO TFT, the simulated transfer curves (*V*_DS_ = 0.1 V), output curves, and the potential drop *V*_XX_ are in good accordance with experimental results (Figures S16–S18, Supporting Information). The mobility is set as 7.5 cm^2^ V^−1^ s^−1^ and subgap states are provided in Supporting Information. Using the same semiconductor mobility but a high electron concentration as 9 × 10^19^ cm^−3^ in the channel (Figure 4a), the transfer and output curves of IGZO‐H TFT could be generally reproduced (Figures 4b,c). The simulated potential drop between the probe A and probe B also follows a rising trend against *V*_GS_ and is much smaller than (*V*_DS_ × *L*_XX_/*L)* (Figure 4d), generally consistent with the GFP measurement. Furthermore, the simulated potentials of the top surface in the channel area are compared with the experimental measurement of SKPM as shown in Figure 4f,g. The merely doped region is only near the contact region regardless of the total channel length, and thus the current enhancement becomes much less significant in short channel devices. The good agreement between simulations and SKPM measurements also supports our proposed mechanism of IGZO‐H TFTs.

Then various film characterizations have been carried out to verify and understand hydrogen doping as shown below. The elemental distributions of the films were examined by secondary ion mass spectrometry (SIMS). To exclude any signal from the encapsulation layer, the SIMS depth profile data were obtained after etching the SiN*_X_*/SiO*_X_* bilayer film. The strongest indium signals from In were used as the reference to which the other signals are normalized, as shown in **Figure** **5**a (see the full signals Figure S19, Supporting Information). The hydrogen signals in IGZO‐H films (black squares) are noticeably stronger as compared with those of the IGZO film (open squares). The different signal intensities suggest that hydrogen atoms have been incorporated into the semiconductor upon deposition and annealing of SIN*_X_*, which is consistent with the above proposed explanation for devices and probably similar to the process employed in fabricating polycrystalline silicon.^21^ A cross‐sectional scanning electron microscope (SEM) image of the films is shown in Figure 5b and the proposed mechanism of hydrogen entering IGZO during annealing is illustrated in Figure 5c.

**Figure 5:**
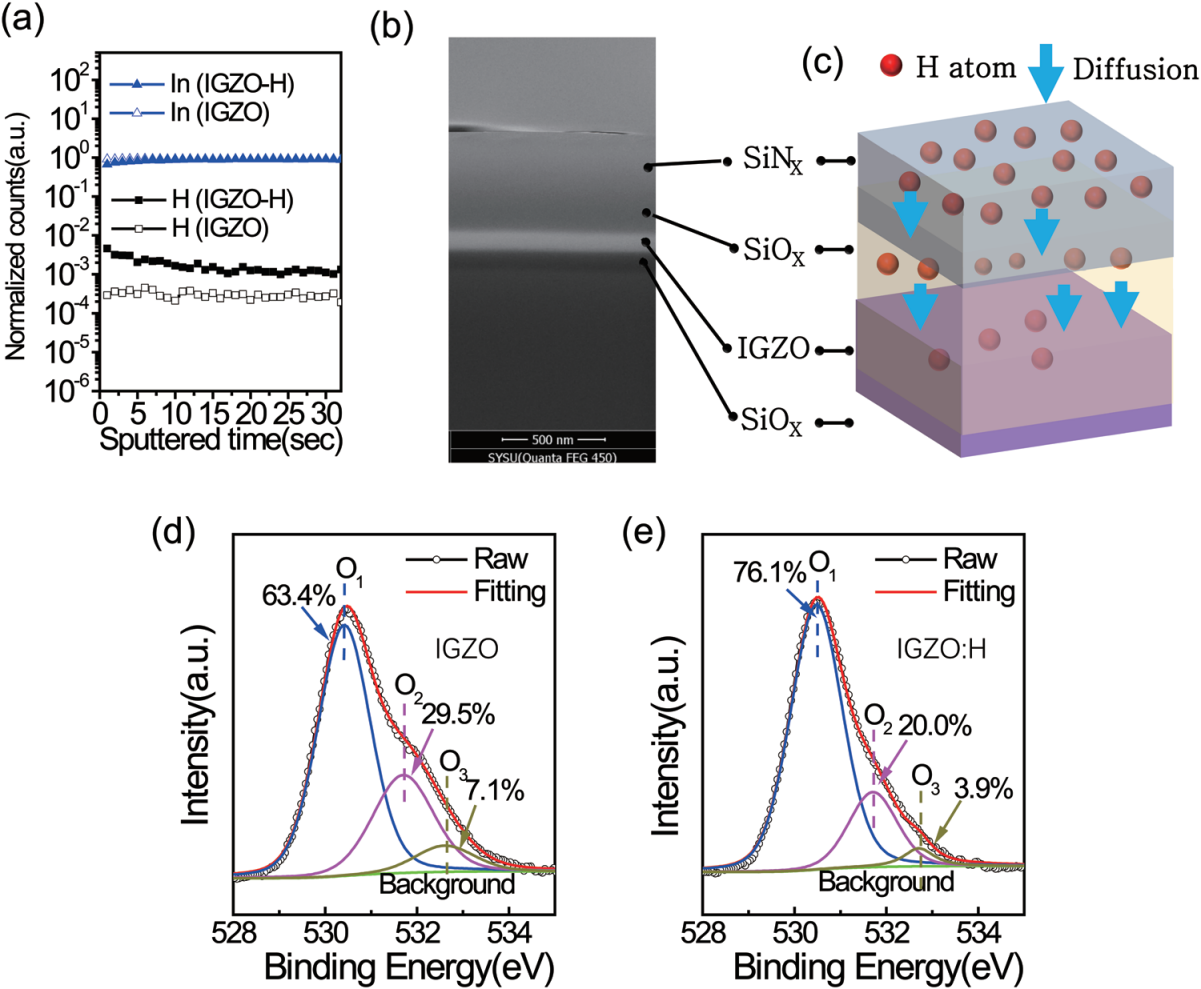
Film characterizations. a) Normalized intensity of the secondary ion mass spectrometry (SIMS) data for the In and H elements for IGZO and IGZO‐H films. b) A cross‐sectional image of an optimized film taken by scanning electron microscope (SEM). c) A schematic view of postannealing process in IGZO thin film. d) Data of X‐ray photoelectron spectroscopy (XPS) for an IGZO film showing the peaks of the oxygen vacancies. e) Data of XPS for an IGZO‐H film showing the peaks of the oxygen vacancies.

The impacts on chemical‐bonding and electronic states are further analyzed by the X‐ray photoelectron spectroscopy (XPS) measurement (Figure 5d,e). The oxygen 1s spectra of film are deconvoluted into three peaks via Lorentzian‐Gaussian fitting with a Shirley type background.^22^ The low binding energy peak (O_1_) centered at 530.4 ± 0.1 eV is related to oxygen ions bonded to metal ions, i.e., In—O, Ga—O, and Zn—O bonds. The middle peak (O_2_) centered at 531.8 ± 0.1 eV is attributed to oxygen defects such as O vacancies (V_O_). The high binding energy peak (O_3_) centered at 532.7 ± 0.1 eV is originated by H_2_O surface contaminants and adsorbed –CO*_X_*.^22^ The ratio of oxygen defects extracted is 29.5% and 20.0% for the IGZO and the IGZO‐H films, respectively. According to previous studies, the reduction of oxygen vacancies is probably related to oxygen vacancies (V_O_) that are substituted by hydrogen atoms.^23^ At the meantime, we also observed a noticeable shift of valence band maximum (VBM) in the IGZO‐H film with respect to the IGZO film in XPS[[qv: 23b,24]] (Figure S20, Supporting Information). Considering that the bandgap (*E*_g_) of both films is almost the same 3.69 eV (see UV absorption spectra in Figure S21, Supporting Information), the shift of VBM implies increased carrier concentration or easier activation of free electrons^25^ in the IGZO‐H film as compared with the pristine IGZO film.

The reduced V_O_ together with the colossally enhanced performance calls into question what role V_O_ and hydrogen ions act as in electron conductivity.^26^ This warrants DFT calculations to achieve a deeper understanding, which provide design rule for both vacuum‐ and solution‐deposited oxide semiconductors.^27^ Calculations were carried out on the a‐IGZO with or without pre‐existed V_O_ and the a‐IGZO with H occupying the oxygen vacancy (V_O_) site (i.e., H‐substitution or H_O_) or interstitial hydrogen (H_S_),^28^ where the methods are described in the Experimental Section.^29^ In the a‐IGZO lattice, the pre‐existed V_O_ has the formation energies of 0.85 eV in O‐poor and 4.76 eV in O‐rich chemical potential limits, respectively (the formation enthalpy of a‐IGZO system is ∆*H*_f_ = −3.91 eV at the ground state temperature). Thus, the pre‐existed V_O_ could be dominant defects due to such low energy cost under O‐poor limit. As the V_O_ will leave two electrons trapped at the nearest neighboring cationic sites, they would act as native point defects for electrons pulling the electronic levels away from the conduction band minimum (CBM). In the a‐IGZO lattice with hydrogen, H_O_ would be mainly discussed in the following as the results of H_S_ are similar (but with slightly higher formation energy).

The calculated electronic orbitals (**Figure** **6**a,b) indicate the hydrogen form multicentered bond with local sites, similar to finding in ZnO and MgO.[[qv: 26b]] The thermodynamic transition levels (TTL) of H_O_ are calculated (Figure 6c). Other related calculated results are shown in Figures S22 and S23 (Supporting Information). The formation energy versus the Fermi level (*E*_F_) shows that the singly positive H_O_ is always lower than the one in the neutral state. Importantly, the reaction of the native point defect with the atomic H, i.e., H + V_O_→H_O_, is found an exothermic process with an effective negative formation heat as −2.85 eV (the zero energy reference state is chosen at the infinitely far from the reactant reservoir based on the Hess Law thermodynamics). Thus, the +1 charge state (H+) can be stable with different Fermi level positions varying through the bandgap.[[qv: 26b]] Also, the H dopant in a‐IGZO in terms of occupying the pre‐existed V_O_ site is expected to be predominantly high in concentration, because the local structural lattice relaxaton would further lower the total energy of the H_O_ even under the O‐rich condition (see the difference of the formaton of neutral H_O_ between unrelaxed and relaxed structural configurations).

**Figure 6:**
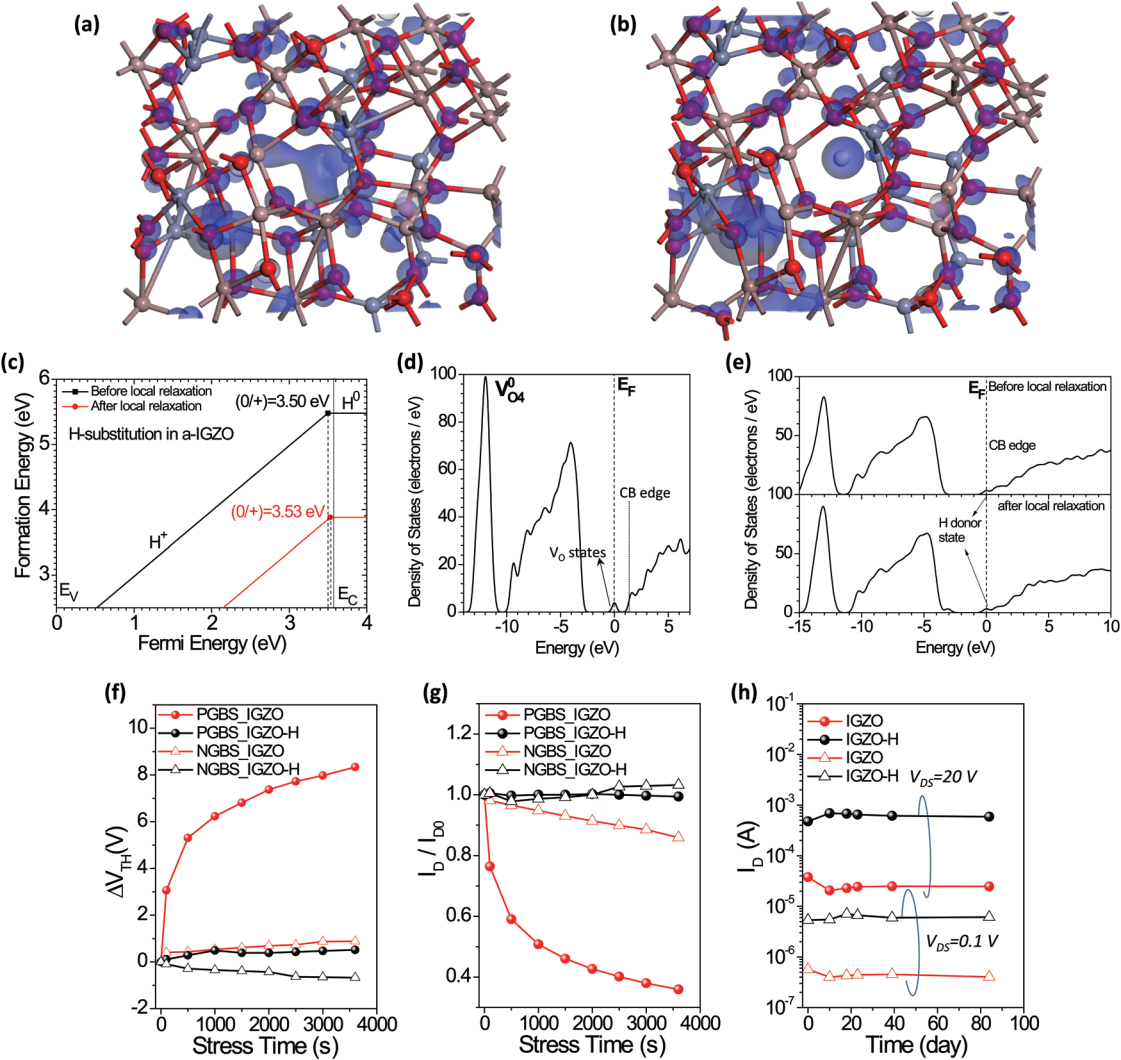
Density‐functional theory (DFT) calculations and reliability tests. a) Calculated electronic orbitals in IGZO, where In = purple, Ga = green, Zn = grey, and O = red. b) Calculated electronic orbitals in IGZO, where H = white. c) The calculated thermodynamic transition level of H‐substitution ions in IGZO before or after local relaxation. d) Density of states (DOS) of the IGZO with oxygen vacancies. e) DOS of the IGZO with H ions substituting oxygen vacancies before or after local relaxation. f) Shift of *V*_TH_ in positive gate‐bias stressing (PGBS) or negative gate‐bias stressing (NGBS) for IGZO‐H TFTs (black dots) or IGZO TFTs (red dots), respectively. g) Evolution of *I*_D_ in PGBS and NGBS (measured at *V*_D_ = 20 V, *V*_GS_ = 20 V). h) Evolution of *I*_D_ in long‐term storage in ambient conditions. The black/red dots are the measured current at linear/saturated regime, respectively.

In total density of states (DOS), the IGZO with fourfold coordinated V_O_ has extra gap‐states about 1 eV from the CB edge (Figure 6d). In contrast, in IGZO‐H with substitutional or interstitial hydrogen, there are no such gap‐states but there are much shallower donor‐like states approaching the CB edge (Figure 6e). From Figure 6c, the TTL of (0/+) for H_O_ stays at (*E*_V_ + 3.50 eV) where *E*_V_ denotes the position of VBM. Considering that the bandgap of the modeled a‐IGZO is 3.54 eV, the ionization energy of H_O_ in donating the electron into CB is rather small, i.e., only several tens of meV. Hence, a small energetic threshold from thermal activation or gate‐field would be expected before transferring into delocalized conducting state with abundant mobile electrons.^13^ This is consistent with our temperature‐dependent measurements which clearly reveal thermally‐activated conductivities (Figure S24, Supporting Information).

Finally, we examine the impact in the device reliability. The results indicate IGZO‐H TFTs are more stable than pristine IGZO TFTs (Figure 6f–h and Figure S5, Supporting Information), as they exhibit a lower extent of *V*_TH_‐shifting and current‐decreasing in positive gate‐bias stressing (PGBS, *V*_GS_ = 20 V) and negative gate‐bias stressing (NGBS, *V*_GS_ = −20 V). After PGBS for 3600 s, the current level decreases to 36% of the original value in regular IGZO TFTs, while it keeps at over 96% in IGZO‐H TFTs (Figure 6f,g). Also, the IGZO‐H TFTs basically possess a long shelf‐life, as demonstrated by the observed small shift of *V*_TH_ when kept in air for tens of days (Figure 6h). The better reliability is attributed to encapsulation and probably hydrogens that reduce active traps in the film.

In conclusion, optimized encapsulation layers of oxide TFTs lead to observation of enhanced current. But such an effect is limited in long channel devices and may lead to misunderstanding of apparent mobility. The origin of enhanced current and on–off ratio is the effective hydrogen‐doping process due to moderate diffusion of hydrogens from the encapsulation layer with postannealing. Gated four‐probe measurements with scaling effect of channel length reveal that the IGZO have been heavily doped in the channel, which is further verified by SKPM and device simulations. By combining the characterizations of elemental profiling, photoelectron spectroscopy, and DFT calculations, we confirm that the enhanced on–off ratio is attributed to the degenerated channel area with stabilized hydrogens. The studies indicate that optimized encapsulation layers can stabilize hydrogens in the channel and lead to enhanced on–off ratio and improved reliability of oxide transistors.

Our study shows clearly that a thorough analysis is necessary to understand the origin of very high apparent mobilities and to avoid the mobility hype in thin‐film transistors or field‐effect transistors with advanced semiconductors, in particular oxide semiconductors. In our case, the real channel length is shorter than what is defined by the source–drain electrode distance as a result of H‐doping of the channel; this causes an artifact in the mobility extraction. However, the mode of device operation identified here is potentially useful: we note that the narrow undoped and resistive regions that are formed near the Mo electrodes are surprisingly reproducible and result in impressive device stability. With some optimization to form even narrower, undoped contact regions this could potentially enable fabrication of high‐performance transistors with self‐aligned and sub‐micrometer channel lengths, that are shorter than what can be defined by the dimensions of several micrometers of standard, top‐down photo‐lithographic techniques used for source–drain electrode patterning.

## Experimental Section

*Fabrication of IGZO‐H Films and TFTs*: All the IGZO TFTs were in a configuration of BGTC. For a regular IGZO TFT, an n‐type heavily doped Si wafer with 105 nm of PECVD‐deposited SiO*_X_* or thermally grown silicon dioxide (SiO_2_) was used as the bottom‐gate electrode and the gate dielectric. The measured capacitance per unit area of the SiO_2_ bottom gate was 32.8 nF cm^−2^. A 70 nm thick IGZO layer was deposited on the wafer at 4 mTorr pressure and, at room temperature using a radio‐frequency sputter with the mixed gas O_2_:Ar = 1:1. The top contacts were fabricated by depositing a Mo layer (200 nm) as the top source and drain electrodes. The channel width and length were 400 and 200 µm, respectively (defined by photo‐lithography). The wet etchant for IGZO and Mo were 1% HCl and a mix of HNO_3_, acetic acid, phosphoric acid, and H_2_O at the ratio of 5:30:120:60, respectively. Finally, the devices were annealed at 350 °C for 1 h in nitrogen atmosphere.

For making IGZO‐H TFTs, a bilayer film with 100 nm of silicon nitride (SiN*_X_*) and 350 nm of SiO*_X_* was deposited using PECVD at 150 °C on top of a pristine IGZO film with electrodes. During the deposition of SiN*_X_* and SiO*_X_*, the chamber pressure was 1.8 Torr and the power was 20 W. In depositing SiN*_X_*, the source gases were 150 sccm SiH_4_/N_2_ (5%/95%), 7 sccm NH_3_, and 400 sccm N_2_. In depositing SiO*_X_*, the source gases were 170 sccm SiH_4_/N_2_ (5%/95%) and 710 sccm N_2_O. Finally, the IGZO‐H TFT was finished by annealing at 350 °C for 1 h in nitrogen atmosphere. Before measurement, the contact holes were photo‐lithographically patterned and etched by reactive ion etching (RIE) at room temperature. During the etching of SiN*_X_* and SiO*_X_*, the power was 200 and 75 W, respectively. In etching SiN*_X_*, the source gases were 5 sccm O_2_, 20 sccm CF_4_, and 50 sccm CHF_3_. In etching SiO*_X_*, the source gases were 25 sccm CHF_3_ and 25 sccm Ar.

*Characterizations of Films and Devices*: The elemental depth profiles were investigated by SIMS. Because there is a capping layer above the IGZO‐H film, the SiN*_X_*/SiO*_X_* films were RIE etched before testing. The SIMS depth profiling of hydrogen, indium, and silicon concentrations was measured in both films. For the XPS measurements, an identical RIE process was applied on the IGZO‐H films before testing. The current–voltage (*I*–*V*) characteristics of IGZO and IGZO‐H TFTs were measured using a semiconductor analyzer (Agilent B1500A) in ambient conditions, except for the temperature‐dependent measurement. It is worth mentioning that the TFT measured by SKPM was built with *L* = 100 µm, thus Figure 4f have been normalized to 200 µm for direct comparison.

*Calculation of Device Parameters and Device Simulations*: Drain current of thin film transistors follows the equation *I*_D_ = (*W*/*L*) *C*_i_μ(*V*_GS_ −*V*_TH_)*V*_D_ in the linear regime where *V*_D_<<*V*_G_. Then linear field‐effect mobility is extracted from transfer curves by: μ_lin_ = (*V*_D_ *LC*_i_/*W*) (∂*I*_D_/∂*V*_GS_), and average value is extracted by the slope of *I*_D_ against *V*_GS_. Linear mobility is calculated as shown in Supporting Information. In saturated regime (*V*_G_<*V*_D_), the current follows *I*_D_ = (*W*/2*L*) *C*_i_μ(*V*_GS_ −*V*_TH_)^2^. Then the saturated field‐effect mobility is extracted by $\mu_{\mathrm{sat}}=(2LC_{i}/W)\;(\partial \sqrt{I_{D}}/\partial V_{\mathrm{GS}})$, and average value is extracted by the slope of $\sqrt{I_{D}}$ against *V*_GS_. For the TCAD 2D device simulations, the details are shown in the Supporting Information.

*DFT Calculations*: 84 atoms were chosen (In_12_Ga_12_Zn_12_O_48_) in an orthorhombic cell with lattice parameters of *a* = 8.03 Å, *b* = 10.51 Å, and *c* = 13.00 Å. And the density was set to be 5.70 g cm^−3^.[[qv: 28b]] The bond ranges from 1.90 to 2.16 Å for Zn—O, from 1.94 to 2.13 Å for Ga—O, and from 2.08 to 2.38 Å for In—O. The general coordination of Zn and Ga sites are from 4 to 6 from tetrahedral to octahedral differently, while the In sites are relatively rigid from 5 to 6 with only octahedral‐like coordination. The O sites are mainly three‐ and fourfold coordinated.[[qv: 28a,b]] For the plan‐wave basis set, the cutoff energy was extended to 750 eV to describe valence orbital components of In^3+^, Ga^3+^, and Zn^2+^, as well as the strongly localized states induced by 2p orbitals of O. To guarantee the convergence and avoid the charge‐spin out‐sync sloshing, the ensemble DFT (EDFT) method was uniformly chosen.^30^ The convergence tolerance of total energy calculations was set to no higher than 5.0 × 10^−7^ eV per atom, and the optimizations of Hellmann–Feynman forces in defect calculations were accomplished to lower than the level of 0.01 eV Å^−1^. The Baldereschi special *k*‐point (¼, ¼, and ¼)^31^ with Gamma‐center‐off was self‐consistently selected. Regarding the geometry relaxation, the algorithm based on Broyden–Fletcher–Goldfarb–Shannon (BFGS) method has been used through all bulk and defect supercell calculations. More details about calculations of electronic DOS and TTL are described in Supporting Information.[[qv: 29b,32]]

## Conflict of Interest

The authors declare no conflict of interest.

## Supporting information

Supplementary

## References

[1] a) A. Bashir , P. H. Wobkenberg , J. Smith , J. M. Ball , G. Adamopoulos , D. D. C. Bradley , T. D. Anthopoulos , Adv. Mater. 2009, 21, 2226;

[2] a) J. F. Wager , Science 2003, 300, 1245; 12764184 10.1126/science.1085276

[3] A. Nathan , S. Lee , S. Jeon , J. Robertson , J. Disp. Technol. 2014, 10, 917.

[4] D. Geng , Y. F. Chen , M. Mativenga , J. Jang , IEEE Electron Device Lett. 2017, 38, 391.

[5] E. Fortunato , P. Barquinha , R. Martins , Adv. Mater. 2012, 24, 2945. 22573414 10.1002/adma.201103228

[6] H. C. Liu , Y. C. Lai , C. C. Lai , B. S. Wu , H. W. Zan , P. C. Yu , Y. L. Chueh , C. C. Tsai , ACS Appl. Mater. Interfaces 2015, 7, 232. 25485556 10.1021/am5059316

[7] X. Liu , C. Wang , B. Cai , X. Xiao , S. Guo , Z. Fan , J. Li , X. Duan , L. Liao , Nano Lett. 2012, 12, 3596. 22694726 10.1021/nl3012648

[8] S. Lee , J. Shin , J. Jang , Adv. Funct. Mater. 2017, 27, 1604921.

[9] H. W. Zan , W. W. Tsai , C. H. Chen , C. C. Tsai , Adv. Mater. 2011, 23, 4237. 21833994 10.1002/adma.201102530

[10] H. W. Zan , C. C. Yeh , H. F. Meng , C. C. Tsai , L. H. Chen , Adv. Mater. 2012, 24, 3509. 22678659 10.1002/adma.201200683

[11] K. Nomura , H. Ohta , K. Ueda , T. Kamiya , M. Hirano , H. Hosono , Science 2003, 300, 1269. 12764192 10.1126/science.1083212

[12] T. Kamiya , K. Nomura , H. Hosono , Sci. Technol. Adv. Mater. 2010, 11, 044305. 27877346 10.1088/1468-6996/11/4/044305 PMC5090337

[13] S. Lee , K. Ghaffarzadeh , A. Nathan , J. Robertson , S. Jeon , C. Kim , I. H. Song , U. I. Chung , Appl. Phys. Lett. 2011, 98, 203508.

[14] H. H. Choi , K. Cho , C. D. Frisbie , H. Sirringhaus , V. Podzorov , Nat. Mater. 2017, 17, 2. 29255225 10.1038/nmat5035

[15] M. S. Fuhrer , J. Hone , Nat. Nanotechnol. 2013, 8, 146. 23459545 10.1038/nnano.2013.30

[16] a) I. McCulloch , A. Salleo , M. Chabinyc , Science 2016, 352, 1521; 27339971 10.1126/science.aaf9062

[17] S. E. Liu , M. J. Yu , C. Y. Lin , G. T. Ho , C. C. Cheng , C. M. Lai , C. J. Lin , Y. C. King , Y. H. Yeh , IEEE Electron Device Lett. 2011, 32, 161.

[18] a) M. Mativenga , S. An , S. Lee , J. Um , D. Geng , R. K. Mruthyunjaya , G. N. Heiler , T. J. Tredwell , J. Jin , IEEE Trans. Electron Devices 2014, 61, 2106;

[19] J. Na , M. Shin , M.‐K. Joo , J. Huh , Y. Jeong Kim , H. Jong Choi , J. Hyung Shim , G.‐T. Kim , Appl. Phys. Lett. 2014, 104, 233502.

[20] a) A. Suresh , P. Wellenius , V. Baliga , H. Luo , L. M. Lunardi , J. F. Muth , IEEE Electron Device Lett. 2010, 31, 317;

[21] a) L. K. Lam , D. L. Chen , D. G. Ast , Electrochem. Solid‐State Lett. 1999, 2, 140;

[22] B. D. Ahn , J.‐S. Park , K. B. Chung , Appl. Phys. Lett. 2014, 105, 163505.

[23] a) J. Kim , S. Bang , S. Lee , S. Shin , J. Park , H. Seo , H. Jeon , J. Mater. Res. 2012, 27, 2318;

[24] A. Abliz , C. W. Huang , J. L. Wang , L. Xu , L. Liao , X. H. Xiao , W. W. Wu , Z. Y. Fan , C. Z. Jiang , J. C. Li , S. S. Guo , C. S. Liu , T. L. Guo , ACS Appl. Mater. Interfaces 2016, 8, 7862. 26977526 10.1021/acsami.5b10778

[25] S. Yoon , Y. J. Tak , D. H. Yoon , U. H. Choi , J. S. Park , B. D. Ahn , H. J. Kim , ACS Appl. Mater. Interfaces 2014, 6, 13496. 25078328 10.1021/am502571w

[26] a) T. Kamiya , K. Nomura , M. Hirano , H. Hosono , Phys. Status Solidi C 2008, 5, 3098;

[27] a) S. R. Thomas , P. Pattanasattayavong , T. D. Anthopoulos , Chem. Soc. Rev. 2013, 42, 6910; 23770615 10.1039/c3cs35402d

[28] a) H. K. Noh , K. J. Chang , B. Ryu , W. J. Lee , Phys. Rev. B 2011, 84, 115205;

[29] a) B. Huang , Phys. Chem. Chem. Phys. 2016, 18, 13564; 27140724 10.1039/c6cp01747a

[30] N. Marzari , D. Vanderbilt , M. C. Payne , Phys. Rev. Lett. 1997, 79, 1337.

[31] M. Probert , M. Payne , Phys. Rev. B 2003, 67, 075204.

[32] a) V. I. Anisimov , F. Aryasetiawan , A. Lichtenstein , J. Phys.: Condens. Matter 1997, 9, 767;

