# Supplementary material for: Analysis of Ultrahigh Apparent Mobility in Oxide Field‐Effect Transistors
Source: Adv Sci (Weinh). 2019 Jan 25;6(7):1801189. doi: 10.1002/advs.201801189 (PMC6446609; doi:10.1002/advs.201801189)
Supplement: Supplementary file 1 — Supplementary [file ADVS-6-1801189-s001.pdf]

Copyright WILEY-VCH Verlag GmbH & Co. KGaA, 69469 Weinheim, Germany, 2019.

Supporting Information

for *Adv. Sci.,* DOI: 10.1002/advs.201801189

Analysis of Ultrahigh Apparent Mobility in Oxide Field-Effect Transistors

*Changdong Chen, Bo-Ru Yang, Gongtan Li, Hang Zhou, Bolong Huang, Qian Wu, Runze Zhan, Yong-Young Noh, Takeo Minari, Shengdong Zhang, Shaozhi Deng, Henning Sirringhaus, and Chuan Liu**

Copyright WILEY-VCH Verlag GmbH & Co. KGaA, 69469 Weinheim, Germany, 2019.

Supporting Information

Analysis of ultra-high apparent mobility in oxide field-effect transistor

Changdong Chen[1#], Bo-Ru Yang[1#], Gongtan Li[1], Hang Zhou[2], Bolong Huang[3], Qian Wu[1], Runze Zhan[1],Yong-Young Noh[4], Takeo Minari[5], Shengdong Zhang[2], Shaozhi Deng[1], Henning Sirringhaus[6], Chuan Liu[1*]

[1] State Key Lab of Opto-Electronic Materials & Technologies, Guangdong Province Key Lab of Display Material and Technology, School of Electronics and Information Technology, Shunde International Joint Research Institute, Sun Yat-Sen University, Guangdong 510275, China
[2] Shenzhen Key Lab of Thin Film Transistor and Advanced Display, Peking University Shenzhen Graduate School, Peking University, Shenzhen 518055, China
[3] Department of Applied Biology and Chemical Technology, the Hong Kong Polytechnic University, Hung Hom, Kowloon, Hong Kong SAR, and The Hong Kong Polytechnic University Shenzhen Research Institute, Shenzhen, China
[4] Department of Energy and Materials Engineering, Dongguk University, 30 Pildong-ro, 1 gil, Jung-gu, Seoul 04620, Republic of Korea
[5] International Center for Materials Nanoarchitectonics (WPI-MANA), National Institute for Materials Science (NIMS), Tsukuba, Ibaraki 305-0044, Japan
[6] Department of Physics, University of Cambridge, CB3 1HK, U.K.

[*] E-mail: liuchuan5@mail.sysu.edu.cn

[#] These authors contribute equally to this work.

Part I. Characterizations of TFTs.

Part II. Reliability test.

Part III. Effects of deposition and annealing of the encapsulation layers.

Part IV. Four-probe experiments and device simulations.

Part V. Film characterizations.

Part VI. DFT calculations of IGZO-H and IGZO.

Part VII. Temperature-dependent measurements.

Part VIII. Mobility comparison with former studies.

Part I. Characterization of TFTs.

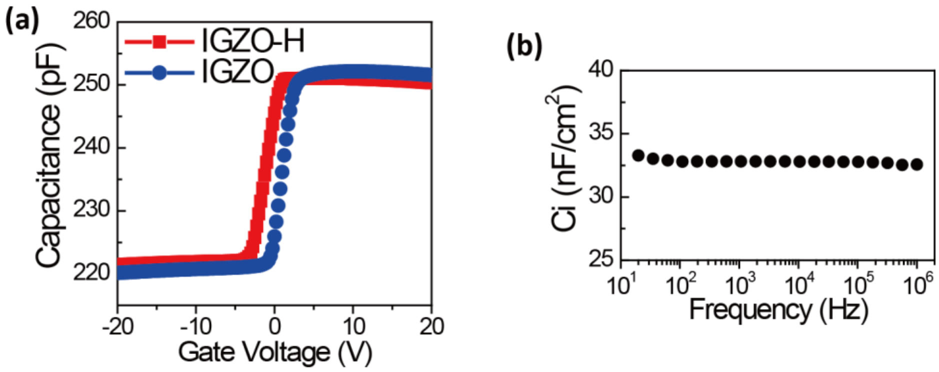

Figure S1 Capacitance measurement in TFT and MIM structure. a. Capacitance-voltage relation for IGZO-H and IGZO TFTs. **b.** Capacitance per unit area of the MIM-structure (Au-SiO_2-Au).

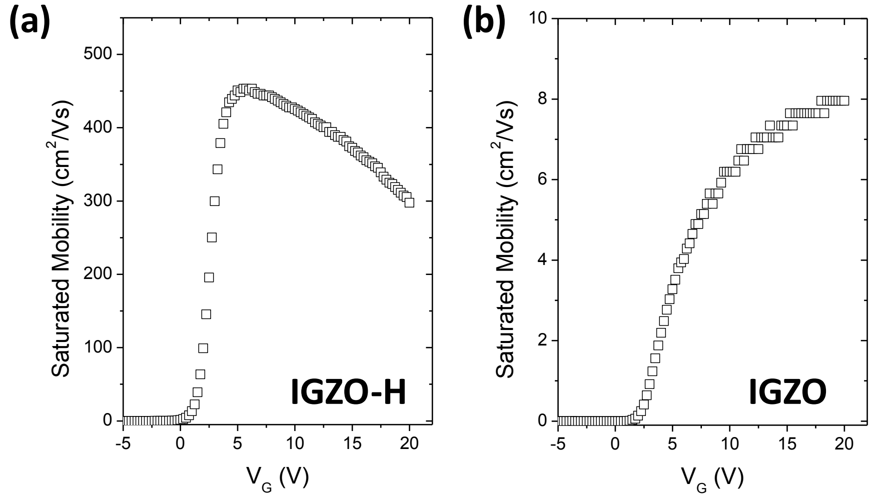

Figure S2. Gate-voltage dependent of mobility in the saturated regime. a. Differential saturated mobility as a function of gate voltage for IGZO-H TFTs. The gradual decreased saturated mobility from 454 cm^2/(V·s) to 300 cm^2/(V·s) could be due to surface or phonon scattering due to strong gate-field, as explained in the reports by Nathan et al.[1] **b.** Differential saturated as a function of gate voltage for IGZO TFTs. The drain voltage is 20 V.

To ensure the mobility values, we examined the extracted mobility by using them to simulate I_D-V_{GS} and I_D-V_D curves as shown below (Figure S3 and S4). The measured I_D-V_{GS} curves are compared with the simulated I_D-V_{GS} curves by using the equation in the saturated regime. In the simulation, the parameters are W = 200 μm, L = 100 μm, C_i = 32.8 nF/cm^2, V_{TH} = 1 V, and μ_{sat}=390 cm^2/Vs. Good fitting are obtained in the saturation behavior in most of the measured window, until there is noticeable deviation when V_{GS}>18V. Hence the extraction of mobility using saturated regime is feasible from I_D-V_{GS} curves (the average slope is used in the main text). The deviation at high V_{GS} may be caused by surface or phonon scattering as observed in other oxide TFTs. In IGZO-H devices, the contact resistance is not considered and thus the real mobility should be higher, which are already over 20 times higher than regular IGZO TFTs. For output characteristics, in the simulation the parameters are W = 200um, L = 100um, C_i =

32.8 nF/cm^2, and V_{TH} = -3V. The mobility values for simulation is 210, 230, 245, and 253 cm^2/Vs for V_{GS} = 12, 14, 16, and 18 V, respectively. The comparison confirms that the extracted mobility is reasonable as they predict the experimental data of current.

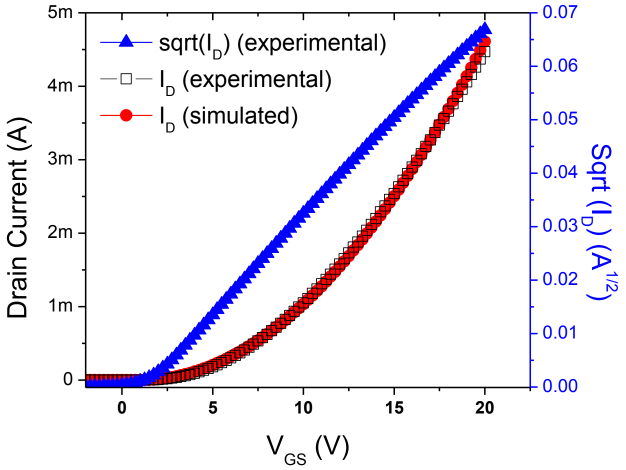

Figure S3. Simulated transfer curves to examine the field-effect mobility. Experimental data of drain current in the saturated regime (open dots) and the simulated data using the saturated regime (red dots).

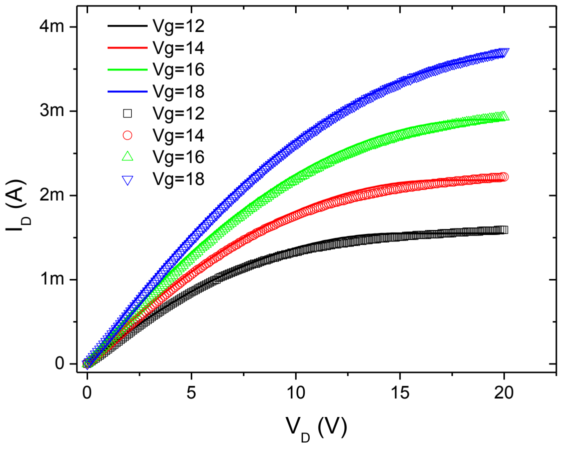

Figure S4. Simulated output curves to examine the field-effect mobility. Output characteristics of IGZO-H TFTs. The experimental data are plotted in open dots and simulation data are plotted in curves (most are within the dots).

Part II. Reliability test.

Reliability tests were given in Figure S5. Summarizing **Figure S5**, IGZO-H films are more stable than IGZO film in stressing and show a long shelf life. The improved reliability of IGZO-H TFTs is attributed to the protection from ambience by encapsulation layer SiO_X/SiN_X and the lower ratio of V_O which induces gradual trapping of mobile electrons[2]. Statistical data are shown in Figure S6.

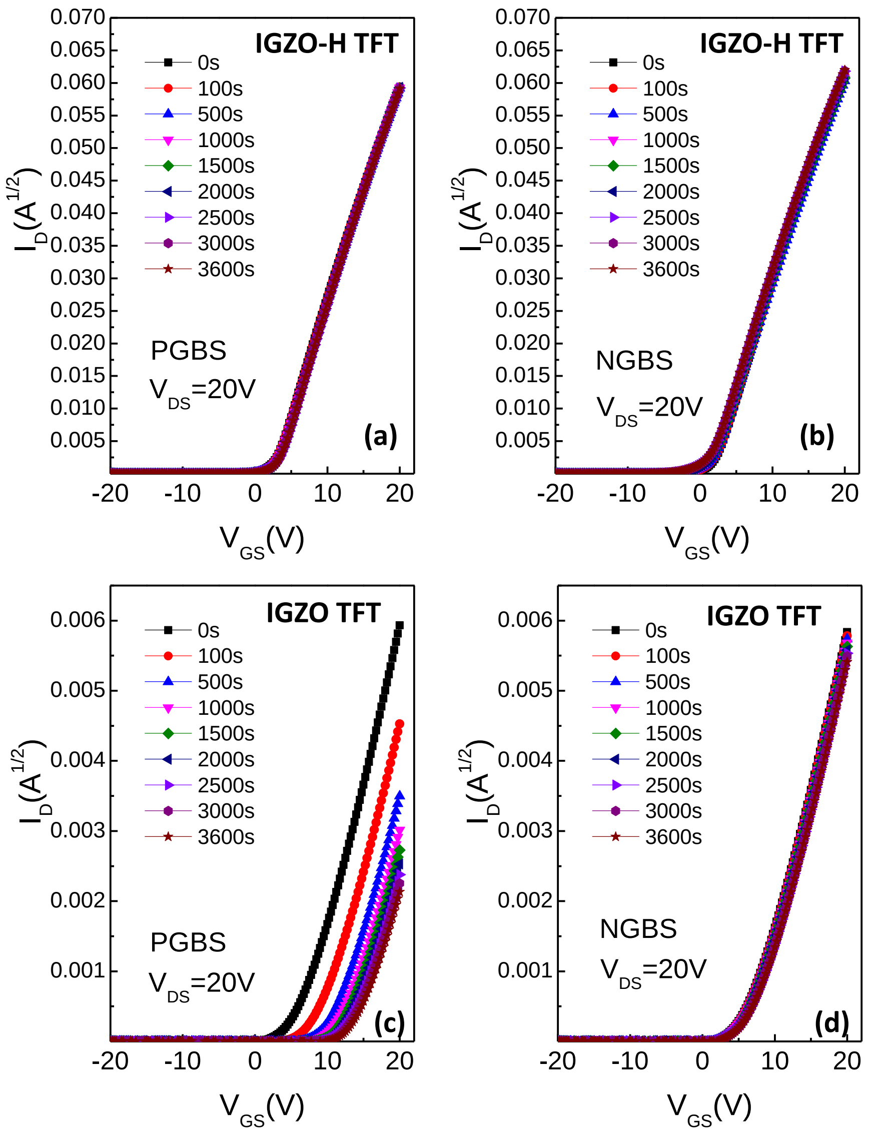

Figure S5. Bias stress testing for IGZO-H TFTs (a,b) and IGZO TFTs (c,d): positive gate-bias stressing (PGBS, V_{GS} = 20 V) and negative gate-bias stressing

(NGBS, V_{GS} = -20 V) Note that square root of I_D is presented to show the linearity with V_{GS}.

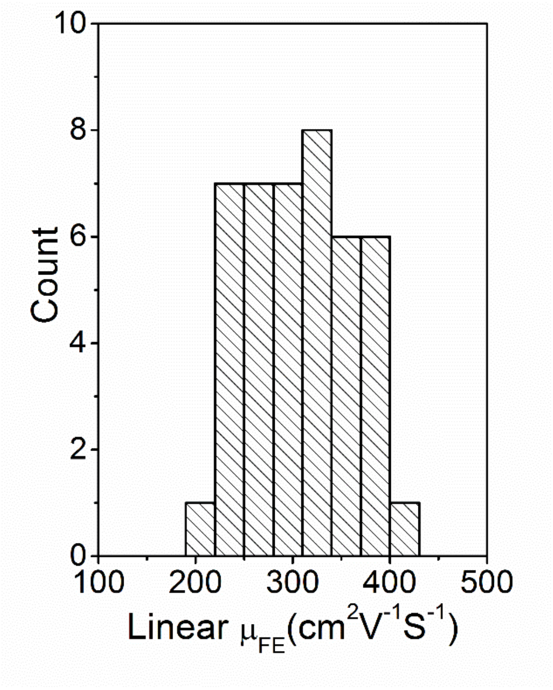

Figure S6. Statistics of apparent field-effect mobility in the linear regime of IGZO-H TFTs in different batches. Statistical information of 43 devices for the field-effect mobility measured in the linear regime.

Part III. Effects of deposition and annealing of the encapsulation layers

The fabrication of high performance TFTs critically relies on depositing and annealing SiN_X/SiO_X layers. The impacts of each layers were investigated as below and the conclusion would be given finally. Moreover, the influence of annealing temperature and time on device performance were studied and presented in this part.

(1) *The effect of SiO_X* is as shown below. From (a) to (b), the results indicate that deposition of SiO_X slightly enhanced current from 4×10^{-5} A to 10^{-4} A

(saturated regime), but the mobility was lowered due to destruction of back channel. From (b) to (c), the results indicate annealing healed the channel and recovered the current and mobility to normal IGZO. Hence SiO_X does not generate H-IGZO transistors with high current.

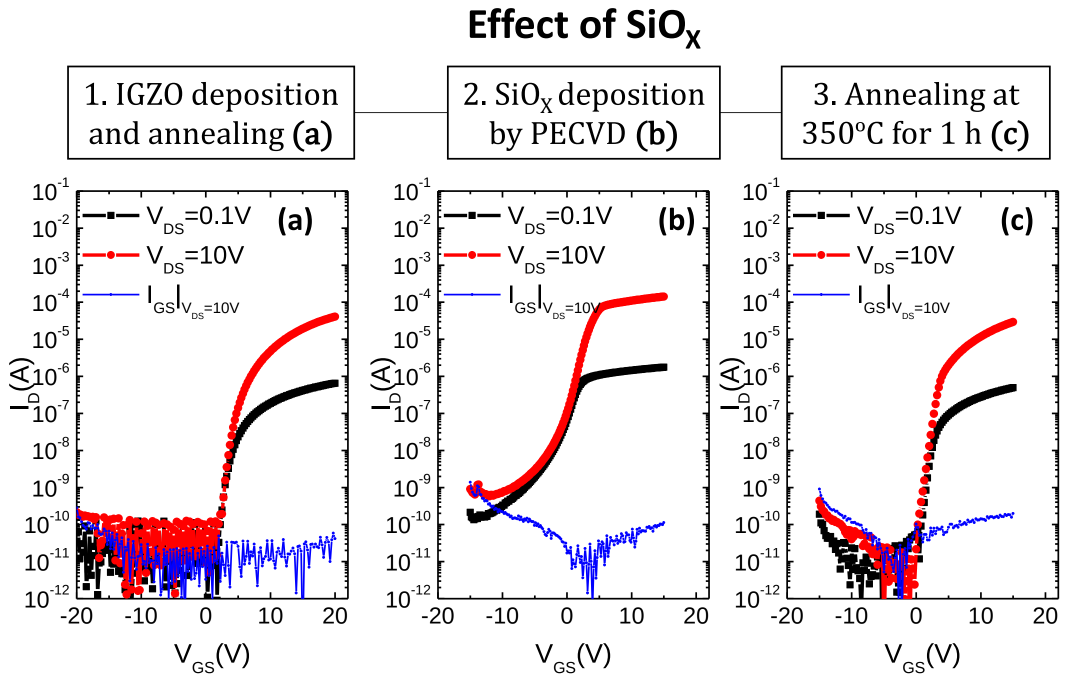

Figure S7. Effect of depositing and annealing SiO_X layer in TFTs. Transfer characteristics of the same TFTs after (a) Deposition of IGZO by sputtering and annealed at 350°C for 1 hour; (b) Deposition of SiO_X by PECVD; (c) Annealing at 350ºC for 1 hour.

(2) *The effect of SiN_X* is as shown below. From (a) to (b), the results indicate that deposition of SiN_X significantly enhanced current from 3×10^{-5} A to 10^{-3} A (saturated regime), and the mobility was enhanced. From (b) to (c), the results indicate annealing heals the channel, maintaining the high mobility of IGZO-H and improves the on-off ratio. But the subthreshold properties were still poor with large *SS* values. Hence it is SiN_X rather than SiO_X that lead to high performance.

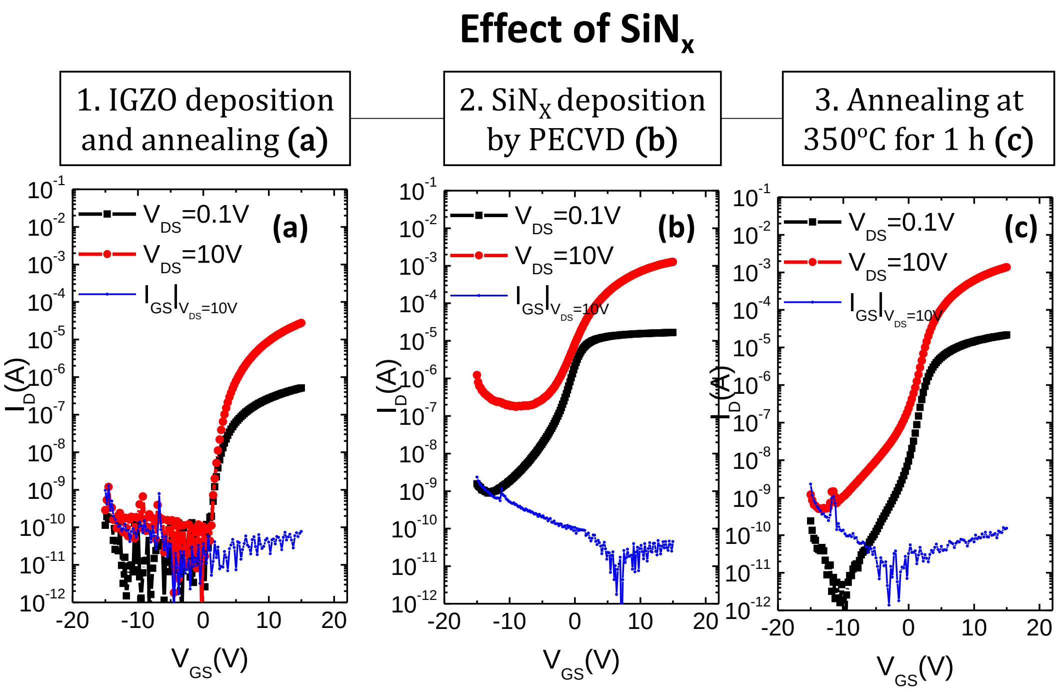

Figure S8. Effect of depositing and annealing SiNX layer in TFTs. Transfer characteristics of the same TFTs after (a) Deposition of IGZO by sputtering and annealing at 350ºC for 1 hour; (b) Deposition of SiNX by PECVD; (c) Annealing at 350ºC for 1 hour.

(3) *The effect of SiNX/SiOX* is as shown below. From (a) to (b), the results indicate that although deposition of SiNX somehow enhanced current from 3x10^{-5} A to 10^{-4} A (saturated regime), the mobility was not enhanced. From (b) to (c), the results indicate annealing heals the channel (step 3), further enhances the current from 10^{-4} A to 10^{-3} A (saturated regime), enables the high mobility of H-IGZO, and improves the on-off ratio. Compared to devices in single SiNX layer (Figure S8), the subthreshold properties were improved with a steeper turn-on behavior. From (c) to (e), the results indicate that once after the final annealing which likely causes the hydrogen diffusion, SiNX/SiOX can be removed without degrading the mobility (step 4). But further annealing again led to mobility that returned to regular IGZO transistors (step 5), probably due to escape of hydrogens from IGZO. Therefore, it is confirmed that SiNX

provides hydrogenation to enable high mobility and protects hydrogen in IGZO-H film, whereas SiO_X is crucial to obtain steep turn-on behaviors probably by protecting IGZO layers.

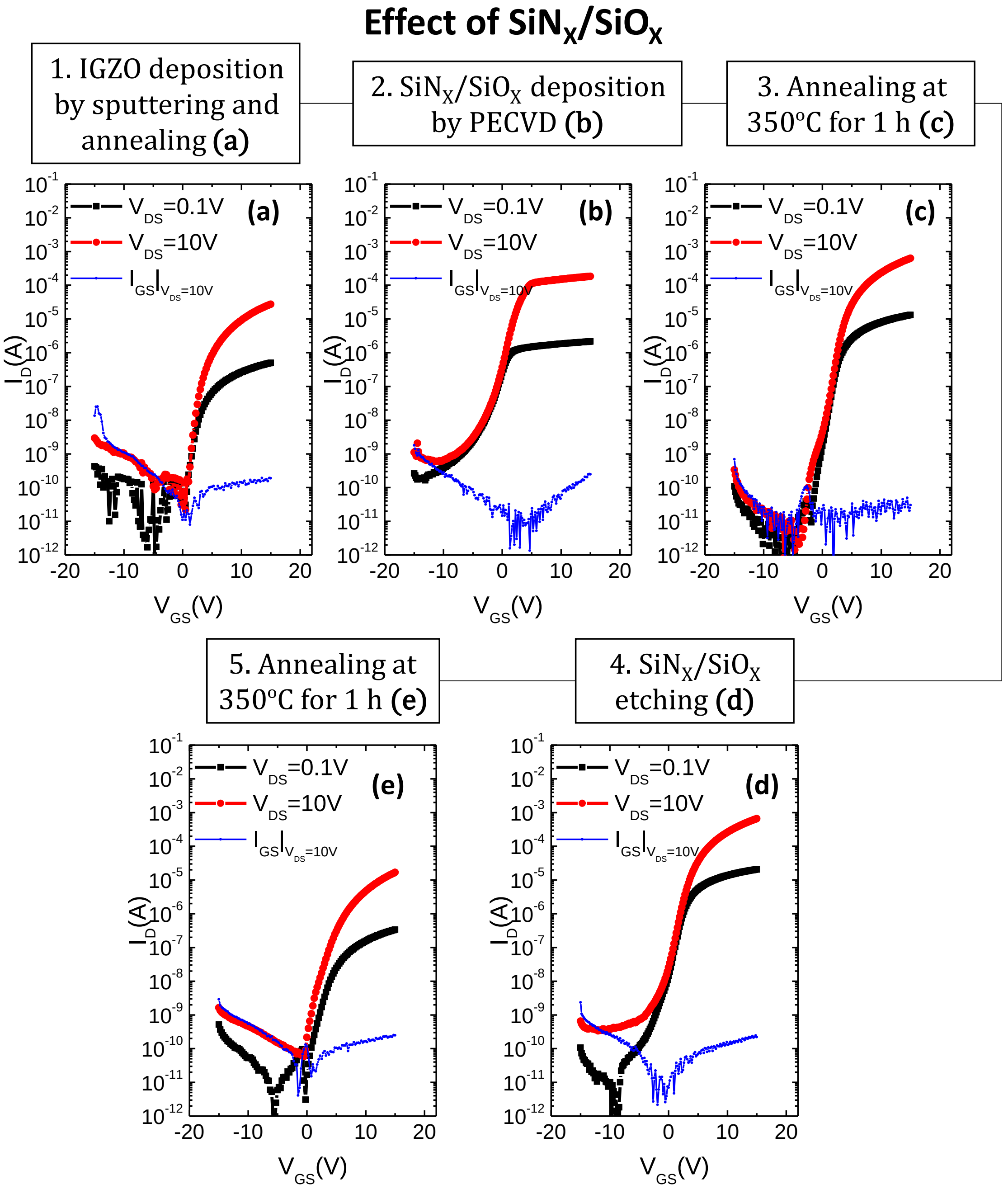

Figure S9. Effect of depositing and annealing SiN_X/SiO_X layer in TFTs. Transfer characteristics of the same TFTs after (a) Deposition of IGZO by sputtering and annealed at 350ºC for 1 hour; (b) Deposition of SiN_X by PECVD; (c) Annealing at 350ºC for 1 hour; (d) Etching of SiN_X/SiO_X; (e) Annealing at 350ºC

for 1 hour.

(4) *The effect of* SiN_X *in annealing* is shown below. From (b) to (c), the results indicate annealing without SiN_X would not lead to high mobility. It suggests that during annealing, SiN_X is critical in providing hydrogen to afford high mobility IGZO-H TFTs.

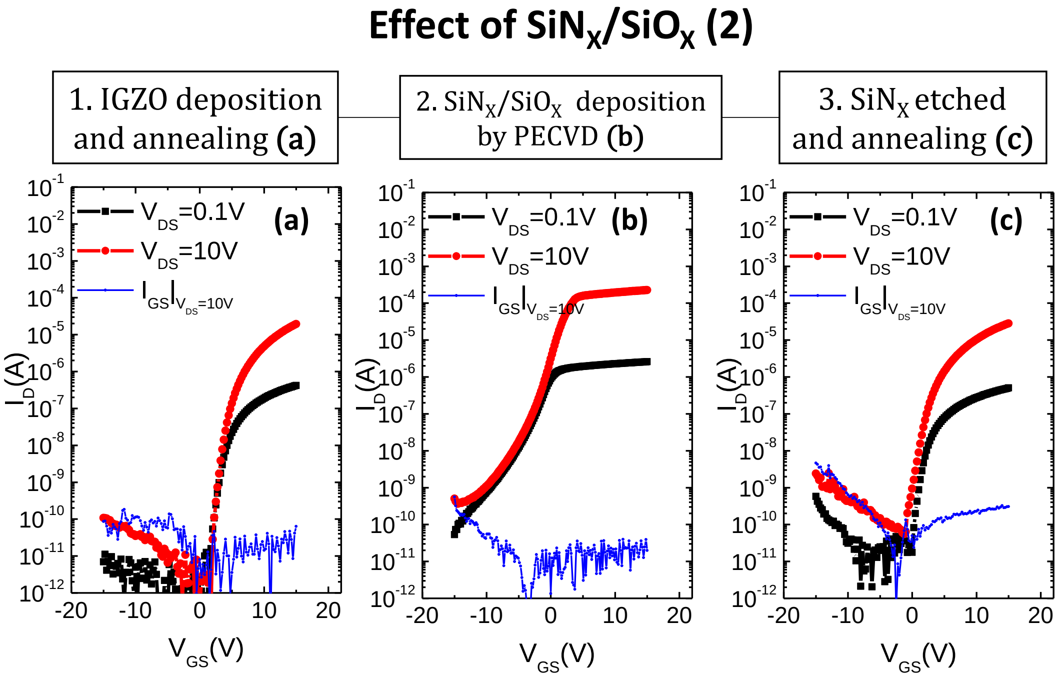

Figure S10. Effect of depositing and annealing SiN_X/SiO_X layer in TFTs (part 2). Transfer characteristics of the TFTs after (a) Deposition of IGZO by sputtering and annealed at 350ºC for 1 hour; (b) Deposition of SiN_X by PECVD; (c) SiN_X etched and annealing at 350ºC for 1 hour.

(5) *The effect of* SiN_X *thickness* is shown below. From (a) to (c), the results indicate that PECVD deposition of SiN_X significantly enhanced current to the similar level regardless of the deposition thickness or time. From (d) to (f), the results indicate the thickness of SiN_X would affect the hydrogenation of IGZO and different mobility and the on-off ratio in transistors. Note that although a

few devices with 300 nm SiN_X maintain can be turned off in the scanning, but most of the devices are conductive as shown in (f). Therefore, it is confirmed the post-annealing rather than PECVD deposition is the most important part in the hydrogenation of IGZO, which leads to high mobility and good on-off ratio. Also, it is possible to control the on-off ratio and mobility by controlling the thickness of SiN_X layer.

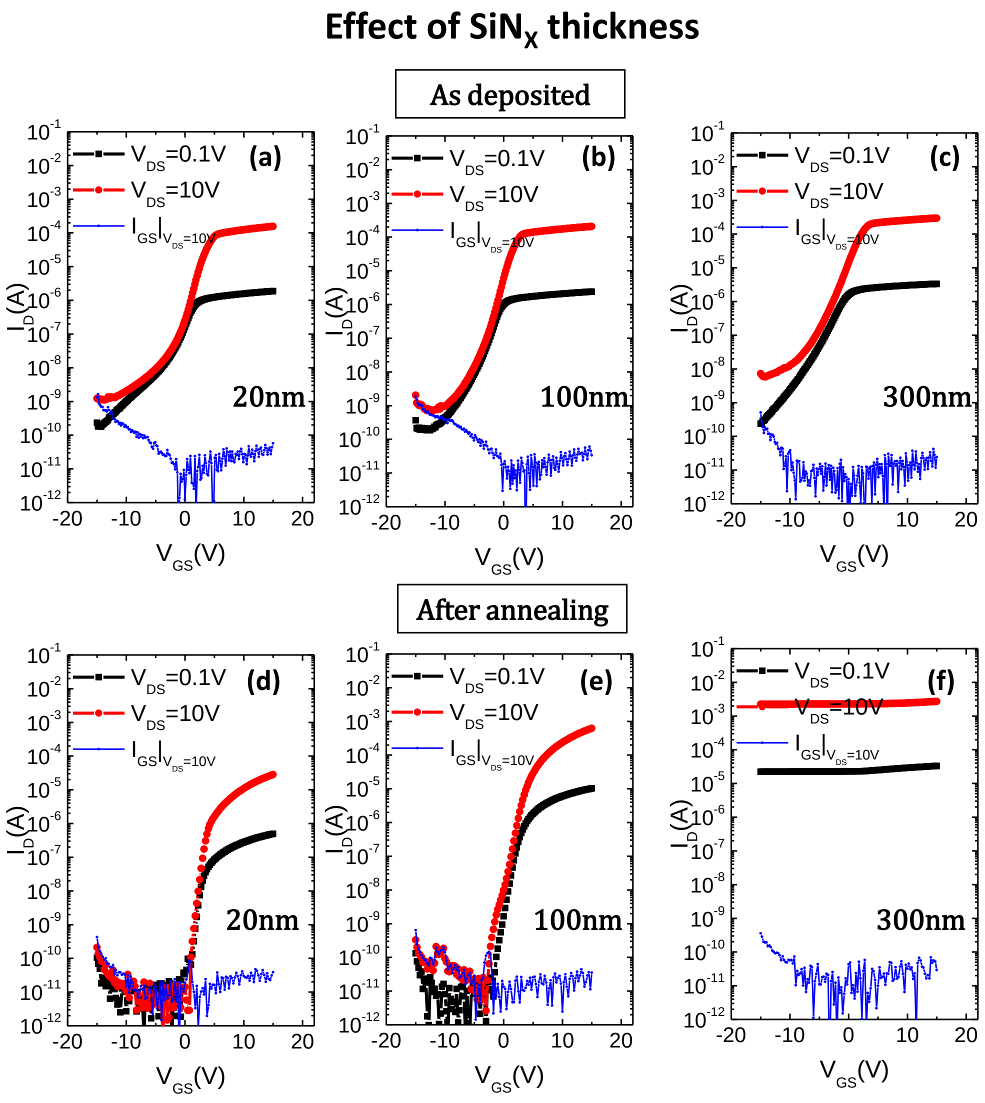

Figure S11. Effect of thickness of SiN_X layer. Transfer characteristics of the TFTs after PECVD deposition of SiN_X in (a) 20 nm, (b) 100 nm, and (c) 300 nm. The same samples were annealing at 350ºC for 1 hour: (d) 20 nm, (e) 100 nm, and (f) 300 nm.

Summarizing the above figures, clearly single SiO_X layer did not affect the current or mobility, whereas single SiN_X enhanced both current and mobility but lead to poor sub-threshold properties with large *SS* values (**Figure S8**). Upon deposition of SiN_X/SiO_X layers on IGZO, the devices did not show high mobility and on-off ratio until after post-annealing (**Figure S9**). In the case that annealing after etching SiN_X, the devices exhibit regular mobility (**Figure S10**). These results suggest that post-annealing with SiN_X layer is the critical step to obtain high mobility; in this process, probably the residual SiH moiety in the SiN_X layer acts as a source of hydrogen atoms and the SiO_X layer acts as a buffer-layer. Finally, the thickness of SiN_X film thickness should be carefully optimized (~100nm) and too thin or too thick SiN_X films led to devices with regular mobility or very low on-off ratio, respectively (**Figure S11**).

(6) *The effect of post-annealing*. We have fabricated IGZO-H TFTs with different extent of hydrogen diffusion by using the same fabrication process but with different post-annealing processes. They were annealed at a certain temperature (200°C, 250°C, 300°C, or 350°C) and tested after being annealed for 20 min, 40 min, or 60 min, respectively. The devices annealed for 60 min are compared in **Figure S12-a**. Only annealing at above 300°C can afford a high current and high on-off ratio. The devices annealed at 300°C for different periods of time are compared in **Figure S12-b**. The on-current show an obvious increase from 5.23 μA to 7.17 μA and to 12.03 μA, with increasing the annealing time from 20 min to 40 min and to 60 min. In comparison, for the devices annealed at 350 °C for different periods of time, the values of on-current are similar and all above 24 μA (**Figure S12-c**).

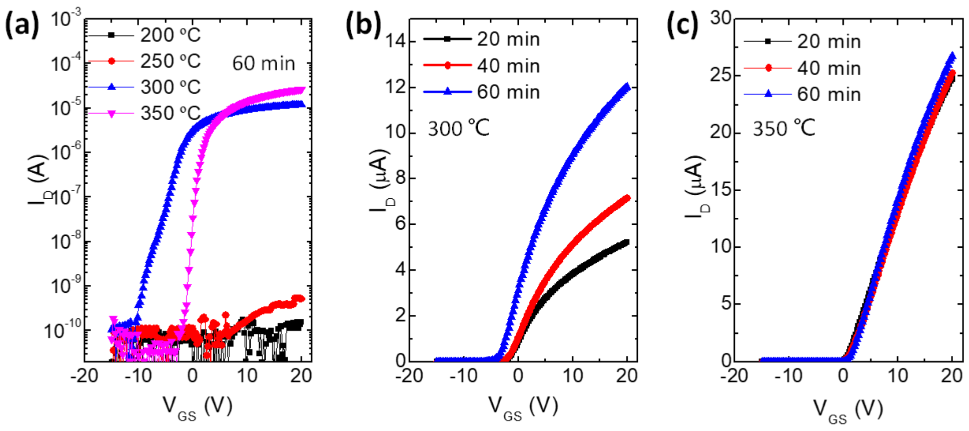

Figure S12. Effect of annealing temperature and time. Transfer characteristics of the TFTs in the linear regime (V_D= 0.1 V) after annealed in (a) 200°C, 250°C, 300°C and 350°C for 60 min of log scale (b) 300°C for 20, 40 and 60 min of linear scale (c) 350°C for 20, 40 and 60 min of linear scale.

The results indicate controllable extent of hydrogen diffusion to vary the device performance. Also, it confirms a suitable post-annealing temperature is necessary to realize a high on-off ratio IGZO:H TFT. And yet we are currently not able to directly detect the amount of hydrogens in the devices, due to limited experimental facilities and to the small mass of hydrogen ions. Then we present the on current of TFTs for the devices with controlled annealing, as shown in **Fig. S13-a and b**.

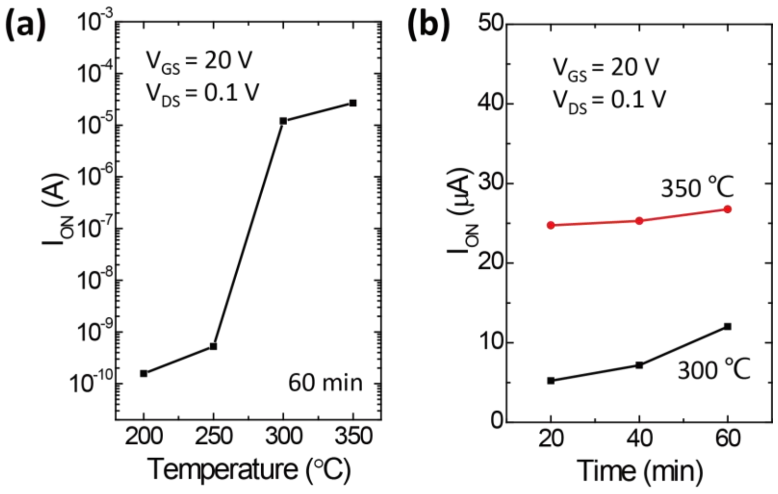

Figure S13. Effect of annealing temperature and time. (a) Device post-annealed at 200°C, 250°C, 300°C or 350°C for 60 min. (b) Device post-annealed at 300°C or 350°C for 20 min, 40 min, or 60 min.

__Part IV. Four-probe experiments and device simulations.__

We have fabricated GFP devices with probes long enough to cross the channel. For IGZO TFTs, the potential of probe A and B saturated at a constant value when the V_{GS} is above 15 V, and the normalized potential drop between each Mo electrode (V_{SA} : V_{AB} : V_{BD} = 0.22 : 0.33 : 0.45) is consistent with the geometry distance (L_{SA} : L_{AB} : L_{BD} = 50 : 70 : 100), as shown in **Figure S14e-g**. In comparison, the potential drops of IGZO-H TFTs (V_{SA} : V_{AB} : V_{BD} = 0.33 : 0.35 : 0.32) are not proportional to the spatial distance (**Figure S15e-g**). Hence, in IGZO-T TFTs, that the channel resistance is mostly decided by the resistance of short, less conducting regions near the contacts.

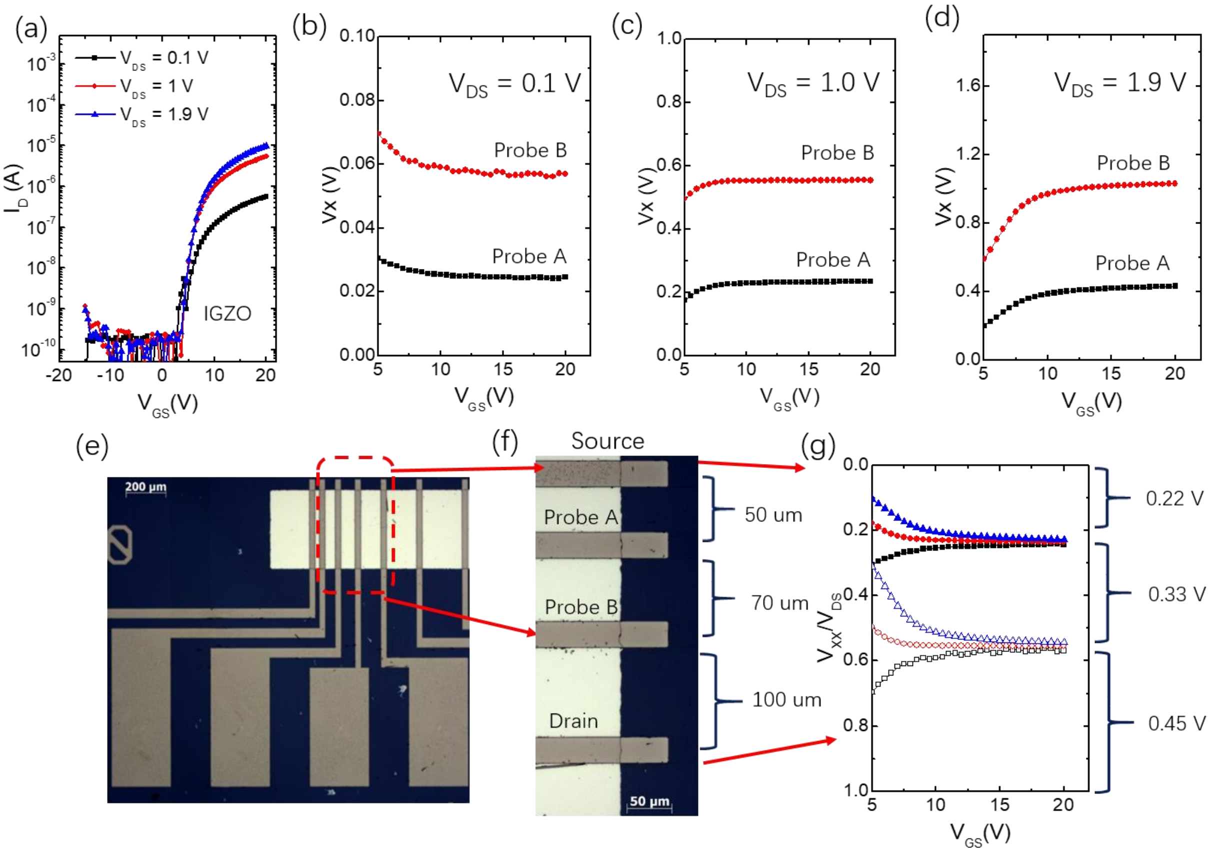

Figure S14 GFP measurements with long inner probes for IGZO TFTs. (a) The transfer curves of IGZO TFTs in GFP Structure. Potential of probe A and B with (b) V_{DS} = 0.1 V, (c) V_{DS} = 1. V, and (d) V_{DS} = 1.9 V. (e) Optical image of one GFP IGZO TFT. (f) The enlarged optical image of the probes and electrodes, and the distance between each two are given in the right. (g) The potential values V_A and

V_B normalized to V_{DS}, and the potential drops between each two probes or electrodes are given in the right.

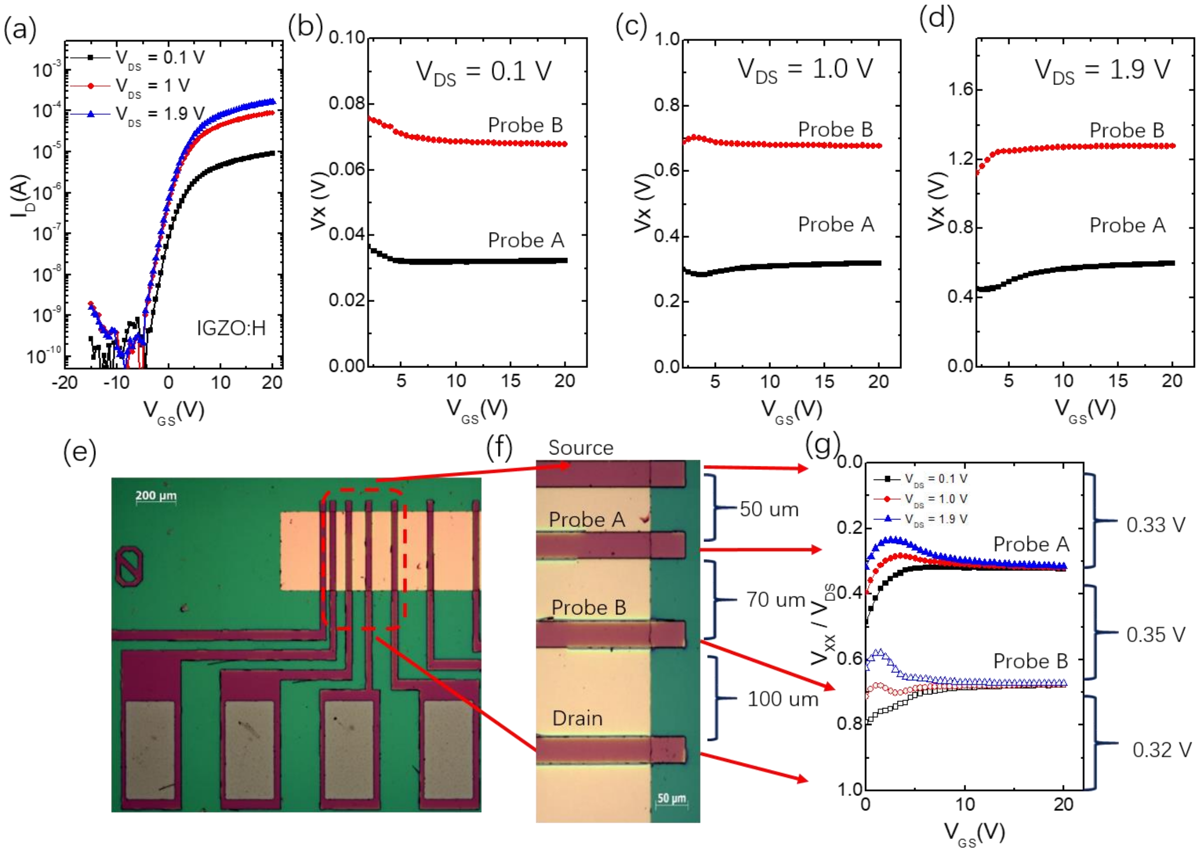

Figure S15 GFP measurements with long inner probes for IGZO-H TFTs. (a) The transfer curves of IGZO-H TFTs in GFP Structure. Potential of probe A and B with (b) V_{DS} = 0.1 V, (c) V_{DS} = 1. V, and (d) V_{DS} = 1.9 V. (e) Optical image of one GFP IGZO-H TFT. (f) The enlarged optical image of the probes and electrodes, and the distance between each two are given in the right. (g) The potential values V_A and V_B normalized to V_{DS}, and the potential drops between each two probes or electrodes are given in the right.

For device simulations, the sub-gap density is simulated using the following model:

$$g_A(E) = g_{TA}(E)+g_{DA}(E) = N_{TA}exp\left(\frac{E-E_C}{KT_{TA}}\right) + N_{DA}exp\left(\frac{E-E_C}{KT_{DA}}\right)$$

$$g_D(E) = g_{TD}(E) + g_{OV}(E) = N_{TD} exp\left(\frac{E_V - E}{KT_{TD}}\right) + N_{OV} exp\left[-\left(\frac{E_{OV} - E}{KT_{OV}}\right)^2\right]$$

The density of states for the sub-gap states are shown in **Figure S16** and the modelling results are plotted in **Figure S17-18**. The calculations were performed as described in [3].

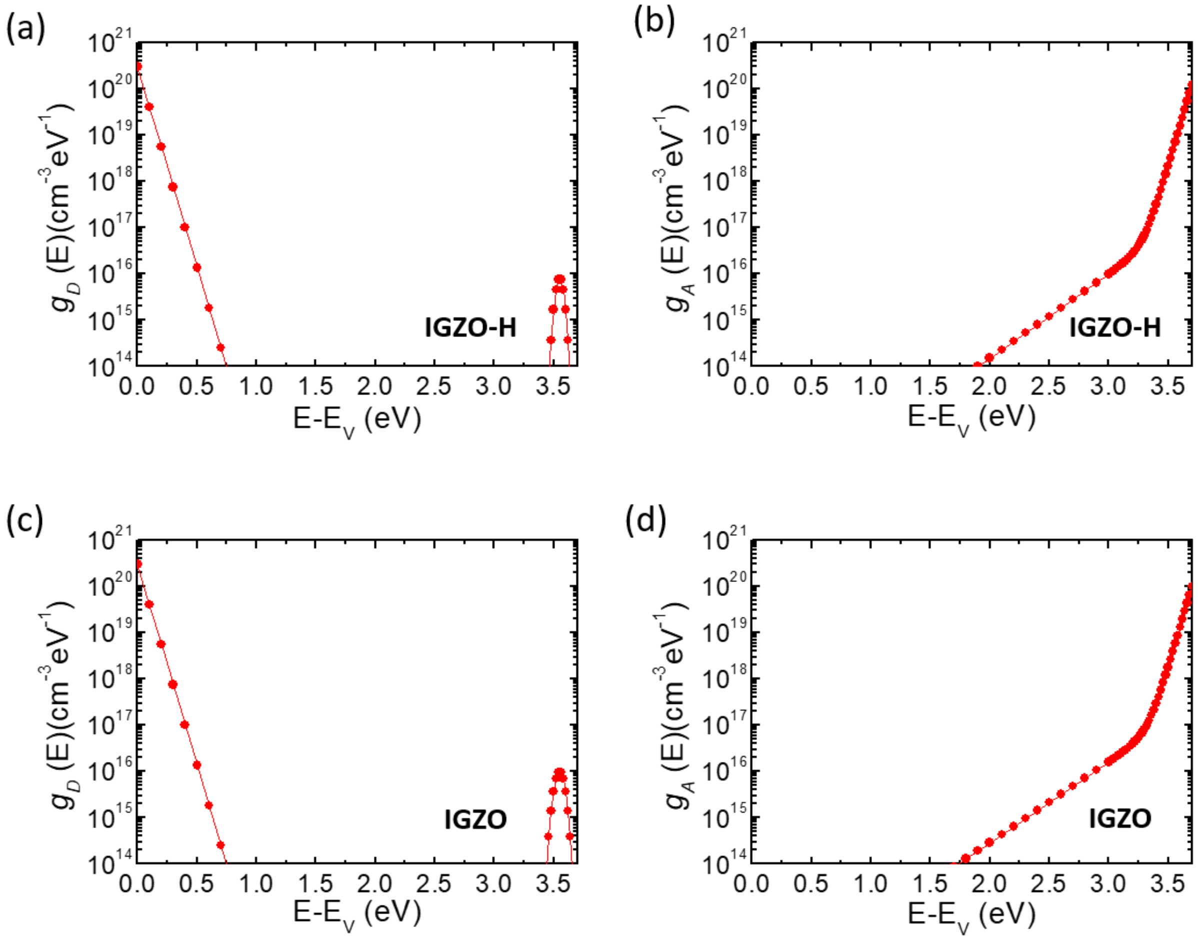

Figure S16 Sub-gap density of state for modelling. (a) Donor state and (b) acceptor state for IGZO-H TFTs. (c,d) are the corresponding data for IGZO TFT.

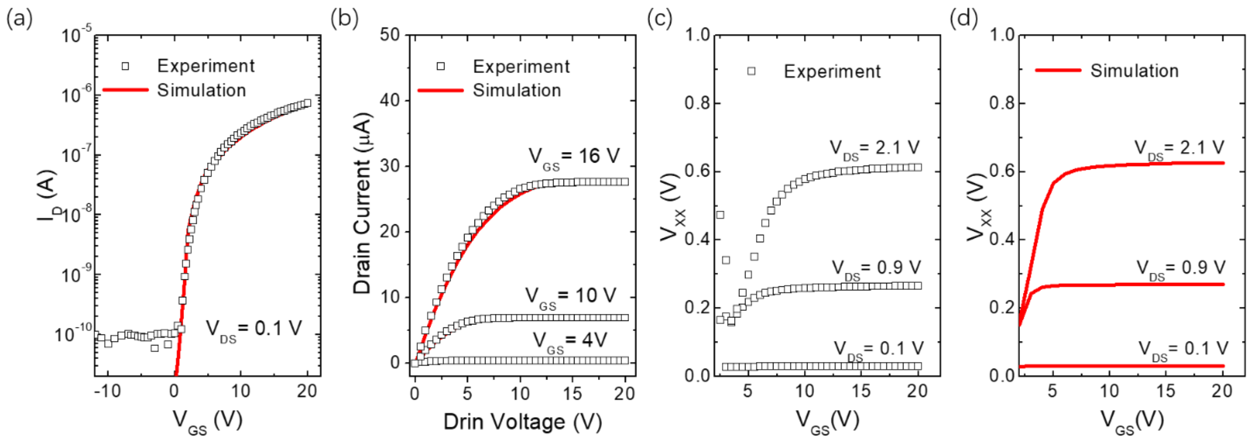

Figure S17 Simulation and experimental results of IGZO TFT. (a) The transfer curves. (b) The output curves. (c) The simulated and (d) the measured potential drop V_{XX} under various V_{DS} values. The dots are experimental results and the curves are modelling results.

For device modelling of IGZO-H TFTs, the doping concentration is 9×10^{19} cm^{-3} and the region under and near the contact (1.8 μm from the source and drain electrodes) is non-doped. The modelling results correspond well to the experimental results (transfer and output characteristics and potential measurements) shown in the main context. For the modelling with other doping parameters (i.e. lower or higher doping concentration) or doping structures (i.e., all channel doped or half channel doped), the results are shown below and significantly deviate from the experimental results.

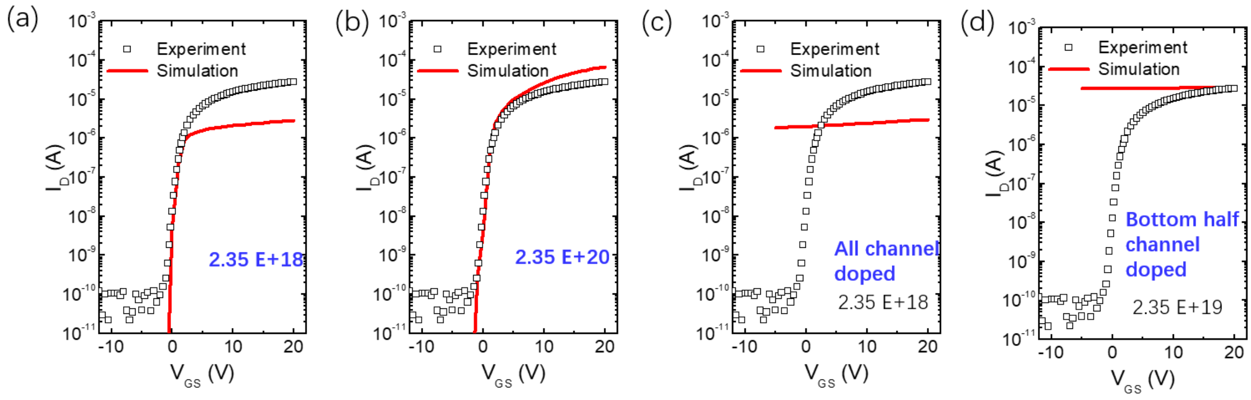

Figure S18 Simulation of IGZO-H TFT with other doping structure. Transfer curves when the carrier concentration of middle IGZO-H area is set as (a) 2.35×10^{18} cm^{-3} and (b) 2.35×10^{18} cm^{-3}. Transfer curves when (c) all channels are doped with electron concentration of 2.35×10^{18} cm^{-3} and (d) the bottom half of channel are doped with a concentration about 2.35×10^{19} cm^{-3}. The dots are experimental results and the curves are modelling results.

Part V. Film characterization.

The film characterizations in elemental distributions and energy band are given.

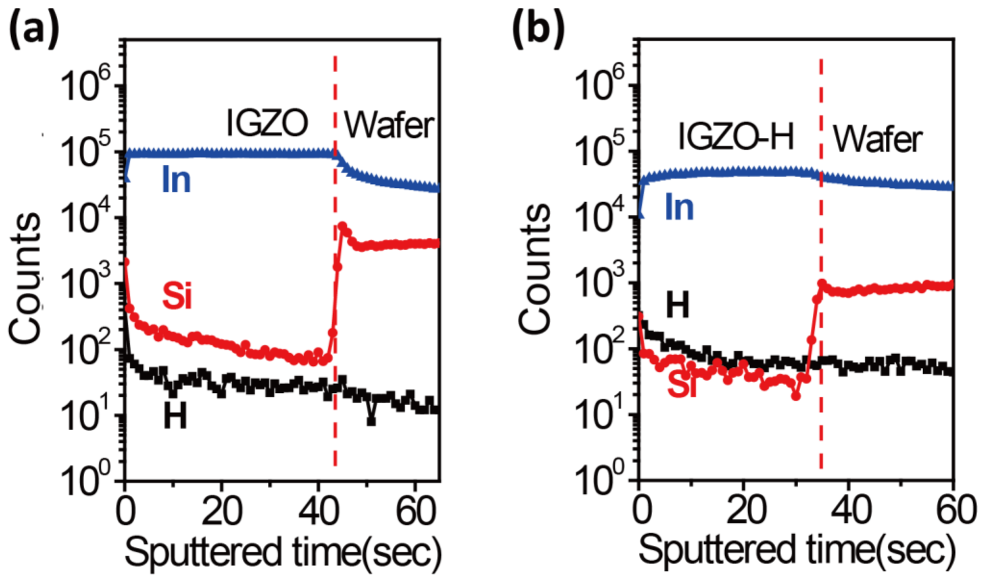

Figure S19. Elemental profiles of the films measured by SIMS for IGZO film (**a**) and IGZO-H film (**b**).

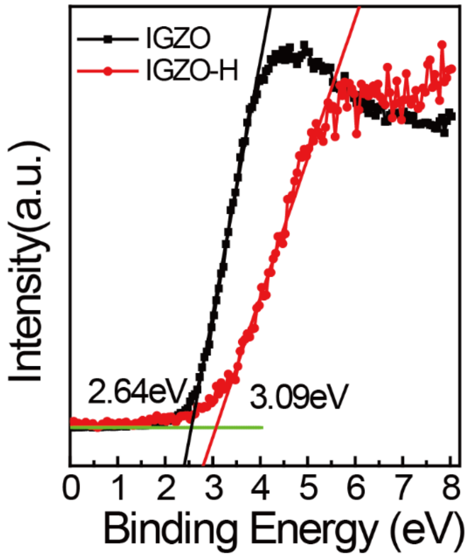

Figure S20. **XPS data** of the valance band maximum for IGZO and IGZO-H films.

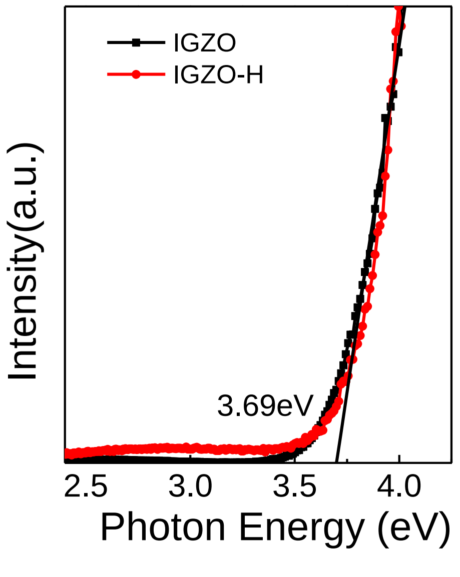

Figure S21. **UV absorption data** for measuring the band-gap for IGZO-H and IGZO films.

Part VI. DFT calculations of IGZO-H and IGZO.

The electronic density of states has been simulated based on anisimov-type rotational invariant GGA+U calculations[4]. By way of OPIUM code in generation with recent RRKJ optimization method[5], we gave the norm-conserving pseudopotentials within the most popular KB (Kleinman-Bylander) projector framework[6], and the non-linear partial core corrections[4a, 7].

Thermodynamic Transition Level (TTL) is the energy level obtained from the DFT calculations. The formation energy of a given point defect at different charge states (q) is calculated at first. The overall supercell was established and remained constantly based on the ground state relaxed primitive cell, in order to avoid the thermodynamic effect of enthalpy changes by cell variations. The formation energy of a targeted defect (H_q) at the specific charge state q can be described as a relation of the positions of Fermi energy (E_F) and the chemical potential $\Delta\mu$ of species of defects α, which is shown as follow:

$$H_q(E_F,\mu)=\left[E_q-E_H\right]+q\left(E_V+\Delta E_F\right)+\sum_{\alpha} n_\alpha\left(\mu_\alpha^0+\Delta\mu_\alpha\right) \quad (1)$$

The above E_q and E_H are the total energy of a relaxed defective lattice in charge state q and the energy of an ideal host lattice at the ground-state, respectively. The ΔE_F in Eq. (1) is the change of Fermi energy with respect to the VBM (setting E_V=0), and n_α is the number of atoms for constituent element α chosen as targeted defect sites, then μ_α^0 is the referenced chemical potential, based on the method used in the work of Zunger et al[8]. The thermal transition energy/level (TTL) $\varepsilon(q/q')$ is the critical Fermi level position in the band gap where the charge state changes from q to q' as ΔE_F changes in the band gap with the lowest-energy, which means the formation energy follows $\Delta H(q, E_F)=\Delta H(q', E_F)$ based on Eq. (1). The detailed forms of TTL have been discussed by Janotti et al[9] and Zunger et al[8, 10] and it is basically calculated by DFT procedure by the following equation:

$$\varepsilon(q/q')=\frac{E_D(q)-E_D(q')}{q'-q} \quad (2)$$

Here $E_D(q)$ and $E_D(q')$ refer to the total energies of the defective structure at the charge state q and q' respectively.

The DFT calculations of IGZO and IGZO with interstitial H ions are given.

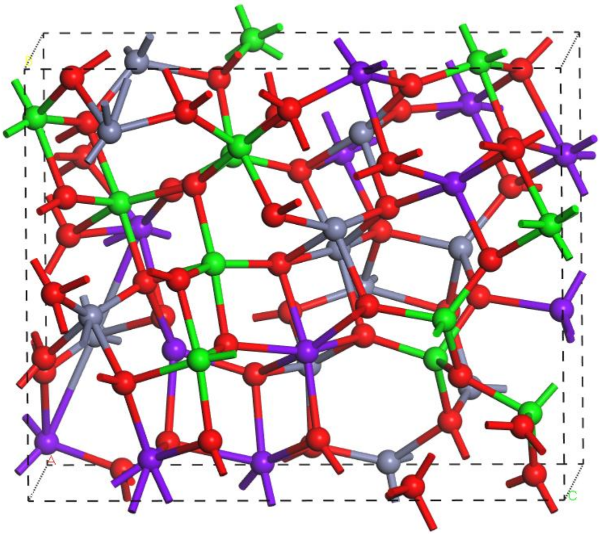

Figure S22. Relaxed structures of the amorphous In-Ga-Zn-O model ($In_{12}Ga_{12}Zn_{12}O_{48}$) (In=purple, Ga=green, Zn=grey, and O=red).

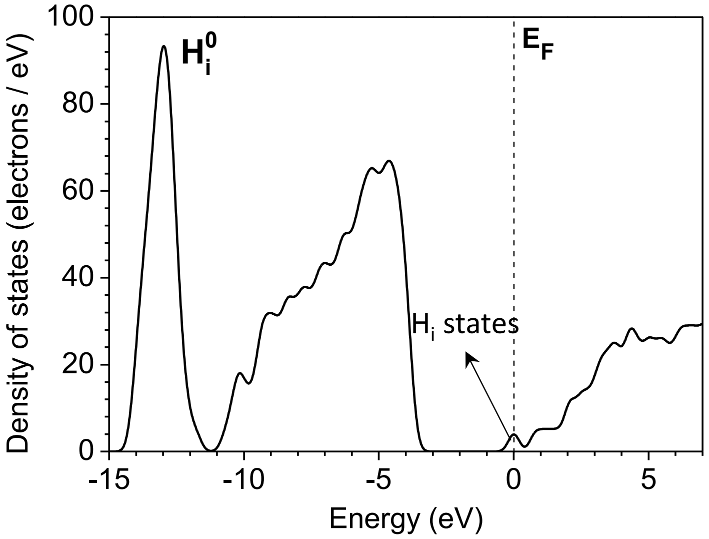

Figure S23. The total density of states of the IGZO with interstitial H ions.

Part VII. Temperature-dependent measurements.

The temperature dependence of conductance $G = \frac{I_\mathrm{D}}{V_\mathrm{D}}$ was measured at around room temperatures (270 K to 320 K) and at varied gate-field (**Figure S24a**). It reveals a thermally activated conduction mechanism, as it follows $G = G_0 \exp\left(-\frac{E_a}{kT}\right)$ (k is Boltzmann constant and E_a is the activation energy measuring the energy difference between the fermi level E_F and the conduction band edge CBM), somehow similar to poly-crystalline silicon[11]. The thermally activated conductivity is apparently related to the donor-like states induced by hydrogen. The values of E_a are plotted against corresponding V_G (**Figure S24c**) and yet the main difference between the two lies in the characteristic conductance G_0, as shown in (**Figure S24d**). The data show that conductivity of IGZO-H TFTs is generally thermally activated.

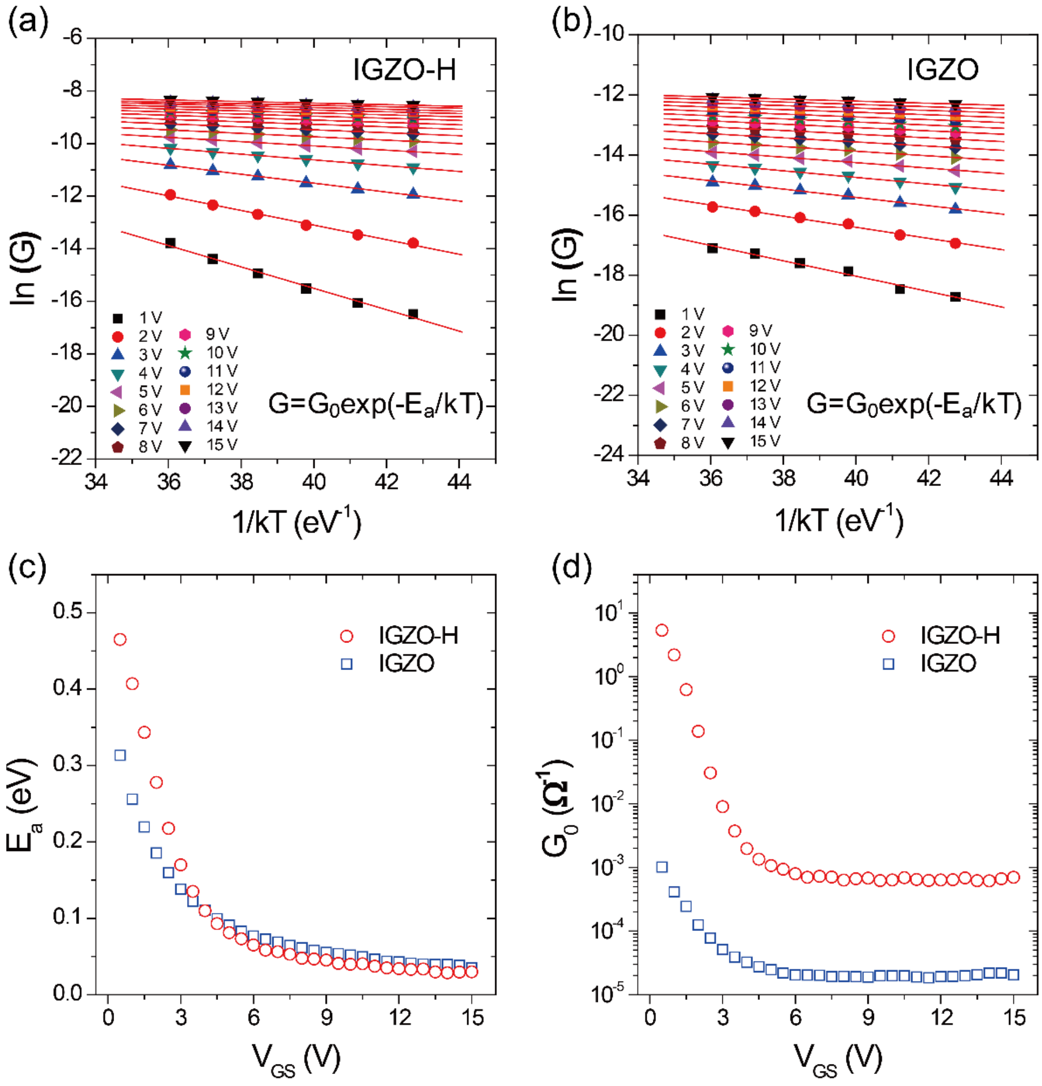

Figure S24. Temperature-dependent measurement of TFTs (from 270 K to 320 K with a step of 10 K, measured in air). The devices were scanned with V_D = 0.1V. Conductance at each V_{GS} for IGZO-H (a) and IGZO (b) devices. Fitting results of $G(T) = G_0 \exp\left(-\frac{E_a}{kT}\right)$ are shown in (c) and (d), plotting the activation energy E_a or G_0 as a function of gate-voltage.

Part VIII. Mobility comparison with former studies.

We summarize our results and compare them with representative results of high mobility TFTs based on IGZO or IGZO composites in **Table S1**. High mobility IGZO TFTs have been frequently obtained by using nano-material composites,

including carbon nanotubes or metal nanowires[12]. Using IGZO itself to achieve high mobility has been accomplished by partially covering the capping layers in the channel, which requires large patterns and a long time (several days) before achieving stable operation[13].. Our approach enables facile fabrication methods and is compatible with conventional fabrication processes.

Table S1 Reported values of high-mobility TFTs based on IGZO or IGZO composites, showing field-effect mobility in the linear regime ("lin") or saturated regime ("sat"). The data shown below are the highest numbers reported in the references and this study.

Method	Width (μm)	Length (μm)	Field-effect mobility $cm^2/(V\cdot s)$	ON-off ratio (saturated regime)	Ref	Year
High K Ta_2O_5 insulator	2000	200	61.5 (lin)	10^5	[14]	2010
Nanometer dot doping	3000	1000	79 (sat)	10^7	[15]	2011
Ca capping IGZO	1000	300	160 (sat)	10^6	[13]	2012
Aligned-SnO2-Nanowire composite	70	20	109 (sat)	NA	[12c]	2014
Silver Nanowire composite	1000	500	174 (sat)	10^7–10^8	[12a]	2015
Defect healing by hydrogens	400	200	345 (lin, ave)<br>392 (lin, peak)<br>402 (sat, ave)<br>453 (sat, peak)	10^7-10^8	This work	2018

References

[1] S. Lee, K. Ghaffarzadeh, A. Nathan, J. Robertson, S. Jeon, C. Kim, I. H. Song, U. I. Chung, *Appl. Phys. Lett.* **2011**, 98, 203508.

[2] M.-G. Kim, H. S. Kim, Y.-G. Ha, J. He, M. G. Kanatzidis, A. Facchetti, T. J. Marks, *J. Am. Chem. Soc.* **2010**, 132, 10352.

[3] G. Li, B.-R. Yang, C. Liu, C.-Y. Lee, Y.-C. Wu, P.-Y. Lu, S. Deng, H.-P. D. Shieh, N. Xu, *IEEE Electron Device Lett.* **2016**, 37, 607.

[4] a) B. Huang, R. Gillen, J. Robertson, *J. Phys. Chem. C* **2014**, 118, 24248; b) V. I. Anisimov, F. Aryasetiawan, A. Lichtenstein, *J. Phys.: Condens. Matter* **1997**, 9, 767.

[5] A. M. Rappe, K. M. Rabe, E. Kaxiras, J. Joannopoulos, *Phys. Rev. B* **1990**, 41, 1227.

[6] L. Kleinman, D. Bylander, *Phys. Rev. Lett.* **1982**, 48, 1425.

[7] S. G. Louie, S. Froyen, M. L. Cohen, *Phys. Rev. B* **1982**, 26, 1738.

[8] S. Lany, A. Zunger, *Phys. Rev. B* **2008**, 78, 235104.

[9] A. Janotti, C. G. Van de Walle, *Phys. Rev. B* **2007**, 76, 165202.

[10] a) S. Lany, A. Zunger, *Phys. Rev. Lett.* **2004**, 93, 156404; b) S. Lany, A. Zunger, *Phys. Rev. B* **2005**, 72, 035215.

[11] a) K. Tsu-Jae, K. C. Saraswat, *IEEE Trans. Electron Devices* **1994**, 41, 1581; b) N. Kubo, N. Kusumoto, T. Inushima, S. Yamazaki, *IEEE Trans. Electron Devices* **1994**, 41, 1876.

[12] a) H. C. Liu, Y. C. Lai, C. C. Lai, B. S. Wu, H. W. Zan, P. C. Yu, Y. L. Chueh, C. C. Tsai, *ACS Appl. Mater. Inter.* **2015**, 7, 232; b) X. Liu, C. Wang, X. Xiao, J. Wang, S. Guo, C. Jiang, W. jong Yu, W. Hu, J. Li, L. Liao, *Appl. Phys. Lett.* **2013**, 103, 223108; c) X. Q. Liu, X. Liu, J. L. Wang, C. N. Liao, X. H. Xiao, S. S. Guo, C. Z. Jiang, Z. Y. Fan, T. Wang, X. S. Chen, W. Lu, W. D. Hu, L. Liao, *Adv. Mater.* **2014**, 26, 7399; d) X. Liu, C. Wang, B. Cai, X. Xiao, S. Guo, Z. Fan, J. Li, X. Duan, L. Liao, *Nano Lett.* **2012**, 12, 3596.

[13] H. W. Zan, C. C. Yeh, H. F. Meng, C. C. Tsai, L. H. Chen, *Adv. Mater.* **2012**, 24, 3509.

[14] C. J. Chiu, S. P. Chang, S. J. Chang, *IEEE Electron Device Lett.* **2010**, 31, 1245.

[15] H. W. Zan, W. W. Tsai, C. H. Chen, C. C. Tsai, *Adv. Mater.* **2011**, 23, 4237.
